# An Historical Essay on the State of the Medical Sciences, during the Last Six Months

**Published:** 1822-07

**Authors:** 


LONDON

### Medical and Physical Journal.

1 OF VOL. XLVIII.] JULY, 1822. [No. 281.

For many fortunate discoveries in medicine, and for the detection of numerous errors, the world is indebted to the rapid circulation of Monthly Journals; and there never existed any work, to which the Faculty, in Europe and America, were under deeper obligations, than to the Medical and Physical Journal of London, now forming a long, but an invaluable, series.—RUSH.

### AN HISTORICAL ESSAY

4630

### ON

### THE STATE OF THE MEDICAL SCIENCES,

### DURING THE LAST SIX MONTHS.

OUR readers will perceive that we have made a slight change in the title of this Essay. On a careful retrospect of the labours of the last six months, we dis over nothing upon which to congratulate ourselves on the progress of our art. It cannot be denied that a certain degree of improvement is making in some of the accessory sciences of medicine; and we think that Pathological Anatomy is cultivated with greater accuracy and success than in former ages. These, however, are so trifling in comparison with the bulk of matter which should pass under examination in a Proëmium of this kind, that it seems more consistent with its object to consider the *state* or *condition* of Medical Literature, including that of the accessory sciences, rather than their *progress*, as heretofore professed.

The first department of this inquiry, that of PRACTICAL MEDICINE, with PATHOLOGY and MATERIA MEDICA, supplies us with proof of the propriety of the change we have ventured to adopt. Surrounded by numerous publications of all dimensions, we have looked in vain for something new in substance or in form. Fortunately, however, we have much that is good and deserving of consideration, to which we can direct our attention.

Among the various subjects which press themselves with equal claims upon our notice, we select *Pulmonary Consumption* as our starting-post: a theme of no ordinary interest, from the fatality which so generally marks its career, and the recent advancements made in our knowledge of its pathology.

For the only solid information we possess respecting it, we are indebted to the talent and the industry of the French physicians: we should rather say, to the opportunities which their excellent institutions afford to the pathological inquirer, from which we are in a great degree excluded by the constitution of our hospitals, and the monopoly of their medical appointments. Hence it is that we find almost every thing that is valuable in morbid anatomy originate with our neighbours; and to us is allotted but the humble task of condensing and arranging the valuable matter they present to our view. We are led to these reflections, seeing that the *pathology of pulmonary consumption*, so ably illustrated by BAYLE and LAENNEC, has occupied the pen of our able countryman, Dr. ABERCROMBIE, of Edinburgh.*

It must have occurred to every practitioner who attends to the apparent progress of medicine, to notice the diversity of opinions which has ever prevailed on the treatment of phthisis. At one and the same time, the very opposite remedies of digitalis and ferruginous medicines were proclaimed as specifics; while some have expressed themselves with entire confidence on the use of the lancet and antiphlogistic measures. Many other discrepancies might be adverted to; the writers invariably losing sight of the fact, that no disease is more varied in its cause. We ourselves have seen it subdued, apparently in its last stage, by salivation, in marked hepatic affection; yet we never dreamt of recommending mercury for its cure under its ordinary form. It is, therefore, of paramount importance to ascertain the various morbid changes to which the symptoms are owing, by which means alone we can ascertain, with precision, the treatment to be pursued, and the probability of effecting a cure. Dr. Abercrombie has rendered, we think, an acceptable service to the profession, by condensing into a narrow focus the information to be desired in this respect. He professes to adduce nothing new or original; but his paper is scarcely the less useful on that account.

The true consumption of the lungs, according to the author, is connected with one or more of the following morbid changes:

The white tubercle.

The semitransparent tubercle.

The fleshy tubercle.

The black degeneration of the lungs, (the *melanosis* of the French writers.)

The hepatized induration of the lungs.

It will be easily conceived that these alterations of structure may remain dormant for a long time, and perhaps through life, without producing symptoms of pulmonary consumption. It is not, however, so generally known that they may go on to a very advanced period, undermining the substance of the lungs, without any clear sign of pectoral disease, the patients gradually wasting with obscure hectic symptoms; the real nature of the complaint being undiscovered, until purulent expectoration appears a short time before death. The cases adduced in elucidation of this fact are very valuable. In one of them, the patient, long before disease of the chest was suspected, had violent and regular paroxysms of fever daily about three o'clock, which were treated as an intermittent, yet without purulent expectoration, the

* *Edinburgh Medical Journal*, January 1822.

matter not having found its way into the bronchial canals. In another, quoted from NONNIUS, extensive suppuration and destruction of the lungs were found after death, although there had been neither cough nor expectoration. The only symptom had been febrile paroxysms, which assumed the character of a quartan intermittent.

Another question connected with this inquiry is, whether, after tubercular disease has taken on the ulcerative action, the ulcer may be cured; or, in other words, whether true consumption of the lungs is ever cured? Patients, labouring under signs of consumption, have sometimes recovered; but we have often no evidence that the disease had its origin in one of the above-mentioned forms. It might have been sympathetic of irritation without the chest, or a common abscess of the lungs, or disease of the laryngeal or bronchial membrane. It is only by the examination of patients who die from other diseases, after having recovered from consumption, that satisfactory information can be obtained. The author relates some particulars of dissections, which lead him to decide in the affirmative.

The *hepatized induration* is considered to be the most rapidly fatal species of true consumption, when it takes on the suppurative action. It may, however, he observes, continue long, perhaps through life, without passing into any farther state of disease, and without producing much uneasiness; and such masses, accordingly, are met with on dissection in the lungs of persons who have died of other diseases. If it be of greater extent, so as to impede, in any considerable degree, the circulation through the lungs, it may give rise to dyspnœa, and may prove a cause of hydrothorax. But, besides these effects of it, it is liable to pass into ulceration of a peculiarly unhealthy kind, constituting a case of a most dangerous and untractable nature, but differing remarkably from the ordinary tubercular consumption.

The indurated mass, in these cases, appears to differ from the tubercular induration, in being more organized, and consequently more susceptible of inflammatory action; in the inflammatory action being more of an acute kind, and running more rapidly into ulceration. It differs from pulmonary substance in a healthy state, in being affected by inflammatory action from slighter causes; in being more liable, when inflamed, to run into ulceration; and in the ulceration being of a peculiarly unhealthy kind. It usually presents the appearance of an irregular cavity, lined by loose ragged ulceration of the indurated substance itself, as if a piece had been forcibly torn out; while the abscess of the healthy substance is lined by a smooth uniform coating of coagulable lymph.

The inflammatory action may be more or less acute, and more or less extensive. The symptoms, therefore, may vary, both according to the activity and the extent of it, and according to the extent of the indurated mass in which it is seated: in some resembling an ordinary attack of pneumonia; in others being at first slight and obscure, more resembling the commencement of phthisis. In both cases, they usually advance rapidly: in the former, assuming the appearance of pneumonia, of a peculiarly untractable character; in the other, constituting a modification of phthisis of a peculiarly rapid kind, remarkably different from the ordinary tubercular phthisis.

In connexion with the preceding particulars respecting the *true* consumption, to use the Doctor's own term, it may be useful to note the following summary of his opinions respecting the other species:

In the affection which is called *sympathetic cough,* "the prognosis is generally favourable; though, in many cases, the appearances are such as to excite considerable alarm. The treatment must vary according to the cause: in the most common form of it, the tonic treatment is required, with exercise, nourishing diet, and minute attention to the bowels.

"2. The *cough from irritation of the diaphragm,* is perhaps most commonly connected with organic disease in the abdomen; and the prognosis, consequently, will be unfavourable. When the cause is more of an active kind, there is more chance of recovery, as in that which is connected with certain acute affections of the liver.

"3. In the *laryngæal phthisis,* the prognosis will depend very much on the period of the disease. If, by minute attention, the nature of the case be detected at a very early period, and the necessary practice adopted with precision and promptitude, its progress may be arrested in a great proportion of instances. If this period be allowed to go by, the disease is generally hopeless. The remedies, in the first stage, are blood-letting, particularly large and repeated topical bleeding, blistering, issues, spare diet, perfect rest, and regulated temperature. In the advanced stages, it admits only of palliative treatment.

"4. The *disease of the bronchial membrane* may terminate favourably in all its stages, even in the more advanced, when the patient is much reduced, and has very much the appearance of an advanced period of consumption. The remedies, in the early stage, are blood-letting, particularly large topical bleeding, blistering, antimonials, with opiates, digitalis, spare diet, rest, and regulated temperature; in the more advanced stages, tonics, stimulants, the balsams, nourishing diet, exercise in the open air, and change of climate.

"5. The *chronic inflammation of the pleura* may often be arrested at its commencement; in its advanced stages, it is generally hopeless. Much attention is required for detecting its existence; as, from the pulse being in many cases little affected, there is great danger of the pain being considered as muscular, and the period being allowed to go by when it may be treated with success. The remedies are blood-letting, particularly topical bleeding, blistering, issues, spare diet, confinement, and perfect rest.

"6. The *disease of the posterior mediastinum* and *bronchial glands* is generally hopeless. It is, in most cases, combined with tubercular disease of the lungs.

"7. The *abscess from active inflammation* may be suddenly fatal by suffocation; or it may be spit up in immense quantities, and the strength of the patient may sink gradually under the discharge, with all the symptoms of consumption. But it may also terminate favourably, even after a large quantity of purulent matter has been expectorated; the abscess healing gradually, if the lungs are otherwise healthy, as a healthy abscess does in any other part of the body. The treatment in this case must be upon the tonic plan.

"8. *Hæmoptysis* may be an acute disease of lungs otherwise healthy, or it may arise from the bronchial membrane; and, in either case, with reasonable attention, the danger is not great. If it arise from lungs previously diseased, it is likely to be followed by consumption; or it may be suddenly fatal by suffocation."

The cases which Dr. Abercrombie relates or quotes, to show that the paroxysm of an intermittent may be symptomatic of diseased lungs, are curious; but the fact is not of unfrequent occurrence.* Two other examples are related in contemporary Journals. The first is that of a man, of a bilious constitution, who for some days had, from seven to nine o'clock every evening, chills, succeeded by heat, dyspnœa, and cough; and perspiration came on about five in the morning. M. d'Olivera was called in about noon. Finding his patient up, and without fever, his countenance sad, belly without tension, no constipation, urine high-coloured and clear, tongue natural, and respiration free, considered *that he had an intermitting fever to treat*. The symptoms continued three days longer; nothing being taken, but restrictions were imposed as to diet, and the patient was ordered diluting drinks.

On the fourth day, at eleven o'clock in the morning, light shiverings, followed by fever and oppression of the chest, came on; the patient could not lay on his left side without a sense of suffocation, obtuse pain in the chest, with cough and mucous expectoration; a dull sound was perceived on percussion of the left side, the remainder of the chest having a natural sound.

This gentleman now abandoned his notion of intermittent fever, and hastened to attack the pulmonary inflammation, whose existence was so evident: he applied twelve leeches on the side affected, by which relief was obtained. Under similar treatment, the respiration became more free, and the patient recovered.

The second is a case of tertian intermittent, accompanied by pectoral symptoms, related by Dr. RENAULDIN.†—A woman, sixty-three years of age, subject to catarrh, experienced, for several days, a fixed pain in the right side of the chest; which, at length, was accompanied by sanguineous expectoration, and became so acute as to render a large bleeding necessary. On the following day, the pain in the side was diminished; but, at two o'clock the morning after, it returned more violently, with shivering and indescribable anxiety, which lasted eight hours; the hot fit then supervened, and was succeeded by perspiration. The patient was cured by sulphate of quinine.

A treatise on *Croup*, by Dr. DESCRUELLES,‡ and a memoir upon the same subject by Dr. GRIMAUD,§ have recently appeared. The former is a judicious compilation of valuable matter; but the latter is especially entitled to consideration, from the extent of pathological research which it displays. In a preceding memoir, the author has shown

---

* *Journale Generale de Medecine*, Fev. 1822.

† *Journal de Physiologie*, 1821.

‡ *Traité theorique et pratique du Croup, d'apres les Principes de la Doctrine Physiologique.* Par M. J. DESCRUELLES. 8vo. pp. 262. Paris, 1822.

§ *Nature et Anatomie Pathologique du Croup.*—Journal Complementaire, Jan. 1822.

that the mucous membranes could be the seat of two very distinct inflammations; one, which attacks the follicular cryptæ, or mucous glands; the other, the membrane itself. The characteristics of the former, which the author entitles follicular, or *white*, phlegmasia of the mucous membranes, were described, with its sympathies and modes of termination.

The disease in question has not been sufficiently considered in its various relations of seat, nature, and extent of inflammation. It is not, in fact, generally known that inflammation of the *cryptæ*, or mucous follicles of the trachea, constitute croup: nor is it understood why the angina trachealis of adults should be different from the disease bearing the same designation in children;—why the mucous secretion is suspended in the first; while, in the second, a new production is formed, which often obstructs the part of the aerial passage with which it is found in contact;—why, in the one there is redness, and in the other paleness, of the surface affected;—why, in the angina trachealis of adults, the pulse is hard and full, while it is below the natural standard in that of children;—why the course of the first has a continued type, and of the latter remittent;—why, in the latter the pupils are contracted, and in the former dilated;—why the tongue, which is red at its margins and point in adults, is pale, larger, and more voluminous, in children.

Our attention is, also, generally confined to the marks of disorder which occur in the respiratory organs; whereas, dissection shows that other disease exists at the same time, which should be taken into consideration. For instance, the mucous membrane of the cheeks and isthmus of the throat is constantly pale. With close attention, the follicles are discovered in a state of enlargement; at the palate, they are often of the size of millet-seeds. The tongue, which is sensibly increased in breadth and thickness, is covered with a greyish coat, sometimes blackish at the base; and its papillæ, exceeding their ordinary volume, give birth to the cracks which are perceived in it. The colour of the pharyngo œsophagian canal is of a pale grey, and the mucous glands are a little larger than in the healthy state, especially at their upper part. Sometimes a whitish membraniform production, of half a line in thickness, extends downwards into the cavity of the stomach.

Most commonly, the stomach is the seat of an inflammation which attacks the mucous membrane, even along its great curvature. Sometimes it assumes the form of small, narrow red zones, formed by a great number of little specks of the same colour, and joined together so as to constitute an appearance of stars; sometimes they are patches of a red colour, taking different directions: the first seems to be owing to lesion of the villosities which abound in this viscus. In these two cases of erythemoid phlegmasia, the cavity of the stomach contains fluids of different colours, white, yellow, grey, green, and black, according to the intensity of the inflammation. The follicles, which have secreted these fluids, are scarcely visible, because they were inflamed subsequently to the other membrane: but, when they have been first inflamed, we see numerous granulations of different sizes, especially towards the two orifices. The follicular secretion has an appearance of a greyish pulp, of firm consistence. The stomach seems starred, and is of a smaller size than ordinary.

The mucous follicles of the intestines are very visible; sometimes they group one set around the other, and form elliptical, granulous, or ulcerated patches. Sometimes they are irregularly placed over a great extent of the mucous surface; and sometimes, again, so near, that they give to the villous coat an appearance of shagreen. In some cases, they are surrounded with little blood vessels; which are the more evident in consequence of their contrast with the whiteness of these little organs. The orifice on the top of the folliculi is easily perceived, and will admit the point of a pin.

Lumbrici and trichurides are also frequently found; and the mesenteric glands partake of the inflammation.

The appearances in the respiratory organs vary according to the causes and intensity of the disease. When croup is preceded by angina tonsillaris, the tonsils are of an ash-white or yellow colour externally: they are more or less puffed up, and give out, on pressure, a fluid of the same colour. Cut into with the scalpel, they have the appearance of a white hepatized lung, with scarcely any red vessels.

In all cases, we may observe a mucoso-albuminous concretion, of a whitish, greyish, or yellowish colour, sometimes a little green, and more or less adherent to the internal coat of the laryngo-tracheal canal. Sometimes it is streaked or spotted with blood; sometimes confined to the larynx and to one part of the trachea; sometimes it is divided into many portions, and occupies points more or less distant from each other; sometimes it lines the whole cavity, and is prolonged into the bronchial ramifications.

The author has frequently seen blood-vessels in the croupal production, as described by Professor CHAUSSIER. The lungs are frequently found inflamed.

Such are the appearances which the gastro-ærian membrane presents in the croup; but these are not all: for, as the author supposes, in consequence of the connexion between the brain and the great sympathetic nerves, the plexus choroides is constantly found inflamed, greyish or brown, and easy to be torn. This inflammation, which gives rise to a limpid, rosaceous, reddish, or yellow, serous effusion, causing convulsions and death, is accompanied always with softening of some points of the medullary substance.

When the inflammation extends to the arachnoids covering the lobes of the brain, which takes place in suffocating croup, the brain itself becomes inflamed, with softening of its substance; and the mucous membrane of the small intestines is also affected.

These facts, which are proved by numerous dissections, throw considerable light on the phenomena of croup.

Dr. WALKER, of Huddersfield, has communicated to the Medical Repository two papers on *Diseases of the Larynx,** with expressions of surprise that so little has been said upon this subject by any but the

* January and April, 1822.

most modern authors. How far back the term *modern* is intended to be applied we cannot divine, but laryngitis, both acute and chronic, has long since excited a very tolerable share of consideration, and seems to be as well understood by the intelligent part of the profession as other complaints. We are not, however, disposed to quarrel with this attempt to excite attention to an insidious and fatal disease, since the commonest facts would be forgotten, unless repeatedly brought within our view; but we must complain of the slovenly mode in which the thing has been done. He talks of the frequency with which it has been *his* lot to witness idiopathic diseases of the larynx, and the melancholy fatality so common to them: yet, notwithstanding the boast of his experience, no one case is related in such a manner as to be a guide to the practitioner; nor are the symptoms communicated by which the malady can be recognized.

Chronic laryngitis, according to the Doctor, is frequently ushered in by catarrh, "*because* he has witnessed extraordinary benefit from the application of leeches to the larynx, in very obstinate cases, which have baffled every other treatment." A medical friend, he observes, experienced its first indications at a season when catarrhs were frequent; and, so mild were the symptoms at first,—so trivial the pain on pressure,—so slight the alteration of the voice, that it was not by any means manifest that any material disease existed in the larynx, of a nature to excite apprehension. Continued cough, some impediment in the deglutition, and subsequent emaciation and hectic, shortly developed the malignity of this cruel invader; and, though the frequent application of leeches, and the use of emetics, kept the disorder in a state of abeyance for short intervals, it was never permanently relieved." In a few months the patient died. A second case is thus related:—"I have seen it fatal in the case of a clergyman, who was for a long time unconscious of its dangerous tendency, and who afterwards received some relief from change of climate. It proved, however, but temporary; for he afterwards entirely lost his voice, his respiration became stridulous, and the disease ultimately fatal."

The author makes some observations on the occurrence of this disease as a sequela of measles, and on the efficacy of leeches applied to the larynx, where frequent hoarse dry cough and dyspnœa prevail.

In one case of examination after death from rubeola, the author observes, there were visible traces of inflammation of the larynx. "In this, however, there had been a retrocession of the eruption, from premature exposure to cold; and the cough, from the beginning, had been remarkably severe." Now we would earnestly impress upon Dr. Walker, that the inflammations of various organs, which accompany or follow measles, are not produced by cold or retrocession of eruption; but, in nine cases out of ten, by the treatment of old women and practitioners of a similar stamp, who keep the patients in an atmosphere of heated air. Cold air and bleeding are the natural remedies of measles attended with inflammation. It is singular that the disease should be ascribed to the retrocession of the eruption, while, at the same time, the author admits that "*the symptoms were remarkably severe*" before it appeared.

The author suspects that disease of the larynx is a common cause of consumption: there is obviously nothing new in this remark, although it seems to be the result of mere suspicion on his part.

The *Diseases of Tropical Climates* have engaged the attention of Dr. CHISHOLM,* Mr. MARSHALL,† and Dr. JAMES THOMPSON.‡ The work of the former has been already fully noticed, and that of Dr. Marshall will form the subject of analysis in our next Number. The observations of Dr. Thompson are contained in a desultory gossiping letter to the Edinburgh Journal, in which we are favoured with a little of *yaws*, a little of *tetanus*, and something upon *worms*. These animals, it is well known, make great havock among children of tropical climates. The author conceives the mortality may be greatly diminished by the timely administration of remedies; on which account it becomes necessary to establish an accurate diagnosis, which is the more difficult as there is scarcely a disorder which these vermin do not simulate.

The symptoms most commonly present are indigestion, watery stools, vomiting, fetid breath, dull, heavy, bloated countenance, voracious appetite, and the food taken passes quickly. When the proper remedies have been too long omitted, the worm fever ensues, and in the strongest forms, so as to deceive the most experienced; dry burning skin, delirium, breathlessness, cough of a peculiar sound, every symptom of pleurisy and dysentery, convulsions and tetanus; the belly is generally swelled, and gets tympanitic after meals.

On the treatment of *Worms* we meet with nothing worthy of notice. Turpentine and sweet-oil, or castor-oil, the author observes, are an excellent remedy when the worms are situated near the stomach.—Can he be serious in confining its efficacy to these cases? We have before us an instance of the effects of oil of turpentine in the removal of *ascarides*, by Mr. NIELL.§ An ounce and a half of oleum terebinthinæ was given to his patient, which operated powerfully as a cathartic, and brought away an immense number of the worms, dead and alive. Although no remedy, previously tried, had succeeded in destroying these troublesome companions, he was materially relieved, for the space of eleven months, by taking two doses only. The worms, however, again accumulated to an extent sufficient to affect his health, when he was cured by an alterative dose of mercury, to which he was subjected for another complaint. The oxymuriate of mercury, even to salivation, has been long since recommended for the treatment of worms.

Two cases of *Tetanus* are briefly related by Dr. THOMSON: one a *frigore*, removed comparatively without any treatment; and another, *traumatic*, from a puncture in the sole of the foot, proved fatal, in spite of the remedies employed, within twenty hours after the first symptom occurred. The spasms came on while the system was under the influence of

---

* *A Manual of the Climate and Diseases of Tropical Countries, &c.* By COLIN CHISHOLM, M.D. F.R.S. &c. 8vo. pp. 236.

† *Notes on the Medical Topography of the Interior of Ceylon, &c.* By HENRY MARSHALL, Surgeon to the Forces. 8vo. pp. 228.

‡ *Edinburgh Medical Journal*, January 1822.

§ *London Medical Repository*, March 1822.

mercury, which had been given, from the time of the accident, for the purpose of preventing the access of this dreadful disorder. The author promises to relate several cases at length, where salivation had been raised after the appearance of the disease, and yet the termination proved fatal.

The only remedies employed during the very short presence of tetanic symptoms, were the warm bath, with ether, laudanum, and camphor, internally. No mention is made of bleeding, as a part of the therapeutic measures proper to be employed; a circumstance the more remarkable, as many cases are recorded which prove its efficacy, at least as an adjuvant to other remedies.

A case is referred to, which we give in the author's words, to show that little reliance is to be placed on amputation as a preventive. "A fine stout negro, who was thrown by accident under the wheel of a heavily-laden waggon, and sustained a severe compound fracture of the leg. Amputation was performed immediately; and calomel, with frictions, used afterwards. On the fourth day his gums were affected; the salivation increased till the sixth and seventh, when he was seized and expired with locked jaw in a few hours."

Baron LARREY, it may be recollected, confidently advises the removal of the injured part for the cure of the disease, where the nerves have been punctured or lacerated by extraneous substances, or the shattered extremities of broken bones.

Several valuable cases of recovery from tetanus are recorded in recent Numbers of our periodical Journals, in all of which copious depletion was employed, with opium. In some the use of mercury was combined, and considered essential to the cure. In a patient of Mr. BARR, of Kelsyth,* the symptoms arose from contusion of the belly, after receiving the injury seventeen days. The remedies consisted in repeated bleeding, opium, and calomel, and the man was quite recovered in less than a month. Other examples of recovery will be found in our Numbers for December and March last; to which we should add the cases related by Dr. KENNEDY, in the Repository for May and June, one of which proved fatal.

Oil of turpentine was given by the latter gentleman, merely as a purgative, without any view to its specific effect. It has, however, been strongly recommended, and may be worthy of notice.

We have before us an excellent paper, with curious facts, on the subject of partial tetanus, written by Baron LARREY,† which we must pass by with this cursory notice at present, but hope to be able to present it entire to our readers in a future and an early Number.

In the mean time we may observe, that we do not think the author happy in his designation of the disease, at least in one of the cases related. In the first, the only symptoms bearing any analogy with tetanus were convulsions in the muscles of the right face, neck, and arm, on any irritation applied to a wound resulting from an accident, which occasioned the symptoms; but the patient had also paralysis of the left arm, intense fever, delirium, stupor, and general convulsions, pre-

* *Edinburgh Medical Journal,* April 1822.

† *Notice sur un Tetanos Traumatique partiel.*—Journal Compl. March 1822.

ceding death: all of which may be accounted for by the existence of meningeal and cerebral inflammation, as was evident upon an examination of the contents of the cranium.

In the second, indeed, strong tetanic symptoms were present; but intense pains of the head, and exquisite sensibility of the nerves of the head and face, constitute a disease known by the very unclassical term of *Tic Douloureux*. The pains in the head, the author observes, were so intense that the patient could not bear the slightest touch without screams, accompanied by shuddering and convulsive movements. The hair and mustachio of the right side stood on end, and transmitted a sensation of extremely acute pain on the slightest touch, or on the incision of a small number of the hairs, even by the finest scissars.

The resemblance between these symptoms and those of tic douloureux reminds us of a case related by Mr. NESSE HILL,* and which had existed for twenty years, under the care of all sorts of practitioners. The patient had been repeatedly leeched, blistered, and purged. Incisions had been made in her gums and palate; every nerve in her face was divided, or attempted to be; and several of her teeth were drawn at one sitting. She had taken carbonate of iron, arsenic, belladonna, and conium. The two latter, and tar varnish, and soap liniment, with opium and saturnine lotion, were externally applied; but all without effect: when, at length, "upon a minute investigation of her then present state of general health, it appeared that the alvine evacuations had long been in an unhealthy state; sometimes scanty, dark-coloured, and scybalous; at others profuse, acrid, and mottled; her appetite either voracious or the reverse; her tongue furred; pulse feeble and quick; a skin affected alternately with chills and hot flushes; her nights tormenting, and her days but little different. She was first put upon the constant use of the mildest aperients until the evacuations were improved, when a strong decoction of cinchona, infused upon quassia, was regularly taken in as full doses as the stomach would admit; at the same time, a grain and half of pure opium was taken every morning, and repeated at noon when pain was unusually severe; constipation and foul evacuations being guarded against by interposed doses of sulphate of magnesia. Thus some ground was gained, especially as to better nights; but still a new sufferer would have deemed the quantum of pain endured by this aged victim intolerable. It was determined to afford another trial to arsenic. It was begun in doses of three drops of the mineral solution, gradually increasing the dose up to twelve drops three times a-day, omitting the opium, excepting occasionally when severe paroxysms occurred. When arrived at nine drops, she was better, and expressed confidence in the plan. At the end of a month, being free from pain, and the system being evidently under the influence of the arsenic, its further use was discontinued *gradatim*. She continued well for several weeks, until the bowels again became irregular. Then a ptyalismic soreness attacked the mouth, the principal seat of the soreness being the part of the mouth first operated upon. Twitchings soon followed, and then a bearable degree of the old evil; but, on regulat-

* *Edinburgh Medical Journal*, April 1822.

ing the bowels, and returning to the solution, all was speedily corrected, and the medicine a second time left off. Three months afterwards, the bowels were much affected with diarrhœa; but no shocks of the old misery returned. Epsom salts removed the complaint; and no further use of the arsenic has yet been required."

Additional cases, illustrating the efficacy of *carbonate of iron* in the cure of this disease, have been communicated by Mr. HUTCHINSON,* and more are promised. One of these patients suffered four or five paroxysms in the course of the day, "generally on washing his face, and whenever he ate or drank. He had long been subject to dyspeptic symptoms, his constitution is leucophlegmatic, and his pulse generally small and languid. Previous to the attack of tic douloureux, he had been subject to very distressing and obstinate chronic inflammatory affections of the membrane lining the trachea. To relieve his painful and truly dreadful attacks of tic douloureux, he has tried a great variety of remedies, and the different modes of treatment recommended by all writers on this afflicting malady. From Mr. Abernethy's judicious system, he certainly derived at one time essential benefit. The solution of arsenic afforded some trifling and temporary relief. The anodyne vegetable extracts have had a long and patient trial, particularly the deadly nightshade, hemlock, and henbane, without producing any material abatement of his torments. When at the sea-side, he has been entirely free from the complaint. At the time he began the use of the carbonate of iron, he was suffering in the most excruciating manner from very frequent and very painful paroxysms of the disease. After the second day of its use, he was considerably better; and in ten days he was not only entirely free from the disease, but in a greatly improved state of health. He has lately experienced very slight attacks, or rather (to use his own words,) such shooting sensations as to induce him to imagine that, were he to commit any irregularities in his manner of living, he might experience some relapse. When the premonitory symptoms occur, he has recourse to the carbonate of iron, which most satisfactorily and speedily removes them. He is now perfectly well."

The disease in the second patient was a painful affection of the nerves of the side, treated in the same manner, with equal success.

Another case of tic doloureux, cured by the sulphate of quinine, is furnished by Mr. PIEDAGNEL.†

A treatise upon some of the diseases of India, under the very absurd name of *Morbus Oryzeus*, has been transmitted to this country from Calcutta, by Dr. TYTLER. The new name has been imposed, upon a notion that the symptoms are invariably occasioned by the employment of new or diseased rice as food. This opinion, it appears, encountered considerable opposition, and even ridicule, from the bulk of practitioners in India. The author may, by possibility, be correct. We have seen repeated instances of grievous disorders produced by diseased corn, or poisonous seeds mixed with corn: for instance, gangrene of the extremities by the seeds of the lolium temulentum. Muscles, also, in this country, and many fish of intertropical climates, possess poisonous

* *Edinburgh Journal*, April 1812. † *Journal de Physiologie*, April 1822.

qualities; but the evidence adduced respecting the rice does not appear to us to be very conclusive. We find, indeed, in every page of the work, the positive assertion of the author; but it is so much the practice of speculators to bend every fact to their own theory, that we are bound to look with extreme caution into statements of this description.

So determined is Dr. Tytler to make rice the cause of cholera, that some children are said to have contracted the complaint by eating sweetmeats, into the composition of which rice entered. Yellow fever, too, if we can believe the author, is also one of the morbi oryzei, because one or two persons had something like it, having, very fortunately for the Doctor's hypothesis, previously partaken of rice for dinner. We propose, however, to give an analysis of the work, which will enable the reader to judge of the value of the evidence adduced. In the mean time we may remark, as a singular circumstance, that, in a later essay,* an attempt nosologically to arrange those endemic diseases of India, which the author supposes to arise from noxious corn or rice, no mention is made of the bad qualities of *new* rice, which constitutes the pervading doctrine of the former treatise.

The author's latest opinions may be fairly collected from the following quotation from the essay in question:

"In consequence of preceding heavy falls of rain, the seeds of the rice crops, which are cut in autumn, and are named in India *Ouse*, *Ballum*, *Patcherry*, *Moongy*, *Sattee*, and *Rarha rice*, and in our price-currents have the various appellations of *coarse rice*, *cargo*, and *yellow Patna rice*, become enlarged in size: their colour changes to black, red, dirty brown, and not uncommonly to a bright yellow or light orange; and a putrid or fetid smell is perceived to be emitted from the bags containing the produce of the autumnal harvest. Immediately beneath the husk, or *paddy-shell*, a thick, oily, and very acrimonious, tunic is found, enveloping the surface of every grain; which covering is named *Kun* by the natives in the Upper Provinces, and *Koora* by those in the Lower. Inferior animals, as well as man, become affected with various diseases, and some of them of the most terrible description, from the employment of grain of this noxious kind as food. In other countries, the farinaceous grains, particularly wheat and rye, are known to be often vitiated, in consequence of the presence of excessive rain, to which they may have been exposed in the course of ripening. But an immense quantity of rice is annually vitiated; and, being introduced into the markets, and disposed of for food, is the general cause and source of some of the most grievous afflictions to which animals, both men and quadrupeds, are liable."

Several valuable essays have appeared on the *Yellow Fever*, by Drs. Archer, Hill, Finch,† Dickson,‡ Rochoux,§ Gerardin,‖

* *London Med. and Phys. Journal*, April 1822.

† *American Medical Recorder*, January 1822.

‡ *Philadelphia Med. and Phys. Journal*, February 1822.

§ *Recherches sur la Fièvre Jaune, et preuves de sa non Contagion dans les Antilles.* Par J. A. Rochoux, &c. 8vo. pp. 452. Paris, 1822.

‖ *De la Fièvre Jaune considerée dans sa Nature et dans ses Rapports, avec les Gouvernements.* Par N. V. A. Gerardin. 8vo. pp. 91. Paris, 1821.

and Mr. COVENTRY.* The symptoms and treatment of this disease are so generally understood, that little novelty can be expected on these points; but we attach a value to the communications, as containing satisfactory evidence of its non-contagious nature. When this shall be generally established, and the yellow fever ceases to be regarded as an imported disease, the only remaining step will be to take measures for its extirpation, which, in our opinion, will follow with as much certainty as the plague has been banished from London by the removal of its fomites. Dr. Hill emphatically remarks, that, when he saw himself day after day entering the loathsome habitations of disease and death, when the full expiration was breathed in his face, and the noisome vapour of the sick man's chamber respired without injury, he could entertain no doubt on the subject, and pursued the duties of his profession without apprehension. He remarked, also, that patients, when removed to healthier districts, did not communicate the disease to others.

The opinion of Mr. Coventry, an intelligent practitioner, who has resided for thirty-five years in the very hot-bed of this and other fevers arising from marsh and putrid miasmata, is entitled to some weight. His paper is wholly devoted to their origin: he says, "The great encroachment made on the river, estimated at ninety acres, composed of perishable or perishing materials, was the evident cause of the mortality at New-York, very similar to what took place last season at Mobile. Blessed with one of the finest and most healthy situations in the known world,—having no marshes of extent that can affect the health in the vicinity,—and abounding on every side with the choicest materials for building and wharfing, the citizens of New-York were inexcusable for using such pernicious stuff to fill their docks, and found their streets on. No city has better opportunities to secure and preserve the health of its inhabitants, than this favoured spot; and it would be the height of madness to permit false theories, and imaginary hypotheses, to preclude the necessity of a strict attention to cleanliness, which is a surer preventive than any quarantine laws that can be devised; for, if the air of a city be as pure as it ought to be kept, although a whole crew from Havannah or the coast of Africa should gain admittance, and die of the yellow fever, you would not hear of any new cases; but the disease would be arrested, the same as it invariably is when the patients are removed to the pure air of the country." In another part he adds, "and the result has been a thorough conviction that the disease denominated yellow fever is epidemic, depends on vegetable putrefaction, and, in that short stage which generally attends the fever, is not contagious; but, if protracted and assuming a typhoid state, as I believe both bilious and inflammatory fevers may and often do, the patient may generate an atmosphere around him, which, without due care, may induce an indisposition in the attendants. But, from what I have observed, I am fully of opinion that yellow fever may be pronounced not to be contagious; for, remove the patient to a healthy and elevated situation, keep him clean and well aired, and I should expect no more danger from this disease than from a tertian intermittent."

* *Edinburgh Medical Journal,* April 1822.

Dr. Dickson is also very full on this point; but it will be unnecessary to make further quotation.

In connexion with the histories of yellow fever, the topography of the situation where it appears, and the local circumstances which accompany it, should be taken especially into consideration. A very full and satisfactory account of these particulars will be found in the essays in question. They confirm the fact that the disease is invariably produced under circumstances favourable to the engendering of marsh and putrid miasmata. We have already adverted to this subject in former Numbers; and have given facts in support of the opinions here maintained. We shall again recur to it more fully, either under the more appropriate head of *Medical Police*, if our space will permit, or in the review of the works of Drs. Rochoux and Gerardin, which is intended for our next.

On the treatment of yellow fever these authors are not entirely agreed; but it will be remarked, that each describes the epidemic of a different country. Dr. Finch carried venesection to a great extent; Dr. Archer prescribed it, with the happiest consequences, in the early stage, if the pulse was full and hard, the countenance flushed, and the respiration impeded; but it could not be safely prescribed after a lapse of twenty-four or thirty-six hours. Dr. Hill thinks the use of the lancet not often demanded in these autumnal diseases. Drs. Dickson and Hill placed the greatest reliance upon mercurial treatment, either by very large doses of calomel or by frictions. It is remarkable that the three first-named authors make no mention of having employed cold-bathing; yet Dr. Archer relates an accidental circumstance which occurred in a patient under his care, as affording additional evidence, if more be wanted, of the utility of cold-bathing in yellow fever. He was an old man, sixty years of age; and, in the third day of the disease, the febrile symptoms ran very high; he had excessive vomiting, and complained of great distress about the head. A cathartic was prescribed; and, in preference to remaining in his chamber, he betook himself to a neighbouring wharf, to wait until it had ceased operating. During its operation he fell into the river, and with difficulty was snatched from a watery grave, by some person passing at the time. He was taken home, and put to bed; a profuse perspiration ensued; his fever left him; and he found himself next day perfectly recovered.

Dr. Dickson says, "The skin was commonly very hot and dry, so much so, indeed, as often to convey to the finger a pungent or burning sensation. To give relief to this, cold bathing was tried, in the different forms of aspersion, affusion, and ablution. The ease thus obtained was very pleasant, though temporary, and the practice was in no instance attended with any bad effects. Whether from its greater convenience, or from the more rapid evaporation producing a greater degree of coolness, it became more general to sponge the body, especially the breast and arms, of the patient, with vinegar or some ardent spirit. Of this last, as a palliative, it is impossible to speak too highly. It substituted, in an instant, for burning heat, the most agreeable coolness; and it appeared very sensibly to relieve the distressing restlessness under which the patient laboured. So much coveted was this very pleasant operation, that I have often heard children cry for it, and adults earnestly entreat for its frequent repetition. I have seen them turn over, after having been gratified, and fall at once into a profound sleep, as if an opiate of irresistible power had been administered."

*Charcoal* is spoken of as an anti-emetic remedy of great value in allaying the irritability of the stomach. In whatever stage of the disease administered, it was attended with the same success; and, even after the appearance of the black vomit, it was rarely that more than the third dose was required to arrest it. It did not invariably prevent the return; but some of the most desperate cases were cured by it, when all other remedies had failed. It appeared to act immediately upon the stomach, by allaying its irritability, and carrying off by stool the black matter, which was found to be perfectly free of odour. The dose was a tea-spoonful every hour or two.

Dr. CHAPMAN, the editor of the Philadelphia Journal, was led to consider the disease as a malignant gastritis, and administered oil of turpentine, as in puerperal fever. Dr. Archer tried it, with "very little advantage."

The plumbi superacet. and the argenti nitras were efficacious in allaying the irritability of the stomach, when other remedies had failed. A capsicum pill was given with the same intention, and often with good effect.

Baron LARREY considers that the yellow fever is not generally contagious, and that it is an inflammatory affection of the peritoneum, especially in that portion which belongs to the biliary apparatus and intestines. He has constantly found more or less fluid effused into the abdominal cavities, with omentum and abdominal viscera inflamed, and covered with gangrenous spots. The liver is equally inflamed, and sometimes contains abscesses within its substance.

After the positive assertions of so many gentlemen of talent and experience in the treatment of yellow fever, relative to its non-contagiousness, it is remarkable that others, of equal talent and consideration, should maintain a different doctrine: yet such is the constitution of the human mind, that the same facts occasionally lead to conclusions diametrically opposite. Thus, the physicians employed to investigate the nature of the same fever in Catalonia, assert it to be highly contagious, and an imported disease.

It may not be generally known that the dreadful ravages of the *Barcelona fever* excited serious apprehensions on the part of the French government, that it might be imported into their dominions; a commission was therefore appointed to investigate, and report upon its nature. Drs. PARISSET, MAZET, FRANÇOIS, BAILLY, and ROCHOUX, were embarked in this undertaking; the three last having had personal experience in the treatment of yellow fever in the West Indies.

Dr. Rochoux, as we have before remarked, was a decided anti-contagionist, and his opinions were not derived without considerable experience, as his morbid examinations, in cases of the yellow fever of the Antilles, sufficiently testify: the others set out with opinions of its contagious nature. Dr. Rochoux, however, very soon altered his notions, and was so struck with the prompt communication of the fever, that he decamped in four days. On the 10th, the party arrived at their destination; on the 12th, the Doctor admitted that, next to rabies, the disease was the most contagious one in existence;* — that the Barcelona fever exhibited the principal characters of the yellow fever of the Antilles, but differed from it essentially by this property of being communicated by contagion; *a fact which now appeared to him incontestible.* A difference was also evident from the danger of the antiphlogistic treatment, so advantageous in the Antilles, but so pernicious at Barcelona. Whether his fears got the better of his reason or not, may become a question: it appears, however, that he very speedily sounded his retreat, leaving to his colleagues the labour and the danger of *proving* the correctness of their opinions by more extended residence among the diseased. This conduct, on the part of the Doctor, might be very prudent; for,

He that fights, and runs away,
May live to fight another day:

but the fact is not very creditable to him, whatever explanation of it may hereafter be given; and it is deserving of notice that, since this event, he has published his treatise on the Non-contagious Nature of the Yellow Fever in the *West Indies.*

The commission, in their Report to his Excellency the Minister of the Interior, labour to show that the local circumstances of Barcelona were not such as have been asserted to exist by their opponents, and enter into some topographical details, into which we cannot follow with any prospect of making them intelligible to such as are unacquainted with the town; or, at least, without putting them in contrast with the contending statements. It forms also a part of their report to account for the manner in which the disease was imported. Many statements are adduced for this purpose; but, as these gentlemen were not present at the commencement of the fever, it is obvious that *their* information must have been derived from others, whose prejudices might have led them to see and to record nothing but that which had a tendency to support their respective opinions.

It seems very clear, from the accounts before us, that the disease did not possess an inflammatory character: but, as we shall resume the subject under circumstances of time and space which will enable us to do it more justice, we shall reserve until then any further particulars.

An account of a *fever*, analogous to that above mentioned, which prevailed among the negroes of Philadelphia, has been read before the Academy of Medicine of Philadelphia, by Dr. Emerson.† As the symptoms were strictly of a bilious remittent, always running rapidly into typhus, he called it *typhoid remittent.* In some respects, he observes, it certainly resembles yellow fever, but a glance at the characteristics will satisfactorily show the distinct nature of the two diseases.

One of the most interesting and singular features of the disease, to use the author's words, were that it selected the blacks for its victims.

---

* *Journale Generale de Medicine*, Avril 1822.
† *Philadelphia Med. and Phys. Journal*, February 1822.

This predilection of a particular form of bilious remitting fever to negro blood, is certainly not impossible; but, from the author's own showing, we think we can clearly discover no foundation for such an opinion in the present case.

Two or three short quotations may be necessary to elucidate this point.

"I can confidently assert that *I* met with no case in a white person, where even equivocal symptoms appeared.

"That *other physicians* may have met with cases in whites very similar to, and perhaps *difficult to discriminate*, from the fever of the blacks, I readily admit.

"The white cases, which I have *inquired* into, were all more protracted, *less fatal*, and evinced many other distinctive marks."

If, then, it should appear that these white cases were the same disease as the black, differing only in intensity, we may perhaps be required to explain the cause of the difference. Dr. Emerson will excuse us for reminding him that the dangerous cases were all on his side. Our readers will readily discern the reason when they hear the account of his practice. We dare not enter into the long detail of his symptoms, but select enough for our purpose in one quotation from among them. "The pain in the occiput, neck, back, and extremities, was greatly aggravated along with the *gastric tenderness and sense of burning*. Such, in many instances, was the *extreme tenderness of the præcordia and abdomen*, that the patient could not bear the gentlest touch upon them without crying out." Now, if ever a physician deserved to be hanged for murder, it would be the one who suffered a malignant *inflammatory* fever, in its marvellous fondness for the negro race, to run away with hundreds of its unhappy victims, without the least attempt to avert the destructive pestilence by bleeding and antiphlogistic treatment. If Dr. Emerson should tell us that bleeding cannot be borne in some of these cases, we answer that, in such, the proper application of cold to the whole surface of the body should have been tried, especially to parts, like the stomach and bowels, suffering under exquisite tenderness and sense of burning.

The following account of the strong marks of inflammation discovered on dissection, renders any further comment unnecessary.

1*st Case.*—Died in two days; the mucous coat of the stomach was *highly inflamed*, as well as the small intestines, both within and without.

2*d.*—Died in four days; appearances the same, though not so great.

3*d.*—Died, after a *protracted* illness, in a typhous state; remains of inflammation in the stomach.

4*th.*—Died on the seventh day; the words of the author are, "Stomach was *also* found inflamed."

5*th.*—Stomach inflamed internally, &c.; duodenum still more inflamed, and the small intestines also participated.

6*th.*—Died on two days; stomach exceedingly inflamed.

The same causes which produce the yellow fever may, under certain modifications, of which we profess to know nothing, produce the

*intermittent.* While the yellow fever, which gave rise to the dissertations just considered, was raging in Norfolk, Surrey, Wilmington, Charleston, and Philadelphia, it appeared also in the neighbourhood of Shuykill, and was *succeeded* by an intermittent; which has given rise to an essay, read, by Dr. HARRIS,* to the Academy of Medicine at Philadelphia. Of five hundred cases of bilious remittent, in each of the years 1820 and 1821, he lost only three patients. From the account of the symptoms and this success, the epidemic was evidently milder than those before described, and free from inflammatory symptoms. The treatment is said to have consisted in emetics of tartarized antimony, cathartics of calomel and jalap, diaphoretics of nitre, tartar emetic, and blisters.

The cases appear to have been convertible into typhus, and into the bilious remittent. In one instance, the paroxysms came on every week,—an hebdomadal ague.

The best mode of treatment is fully detailed. We shall not be expected to notice that part of it by evacuations and bark, which must be familiar to every apothecaries' boy: but it may be well to give an account of the author's experience with some remedies less commonly used, although they are not new.

In 1821, the cases were predisposed to assume a typhoid character. The pale and yellow bark failed, although apparently genuine. A very superior red bark was produced, and every patient, whose stomach retained it, was speedily relieved. It was combined with serpentaria and salt of tartar. When it purged too much, it was combined with laudanum; if vomited, half an ounce was given in an enema, with twenty drops of laudanum, every six hours. This plan was useful in children.

Three children out of four were cured, in about a week, by cataplasms of red bark, bruised garlic, and warm vinegar, applied to the wrists and bottoms of the feet every twenty-four hours. The same benefit was not derived from the same application to the inner part of the arms and thighs; from which he infers that the effect was not produced by absorption, but by sympathy.

Oil of turpentine was useful in typhous cases.

Laudanum, from sixty to one hundred drops, given at the approach of the chilly fit, often prevented another paroxysm; and in no instance failed to relieve the patient of much suffering.

Arsenic was considered a medicine of great value.

The dogwood (*cornus florida*, or *circinata*,) was highly esteemed.

Cobweb was tried, but without success. One patient swallowed a black spider in an entire state: it occasioned extreme nausea, delirium, and vertigo, until he vomited it on the third day. It had no effect in curing the disease.

Sulphur was given with advantage. Cures by it are said to be less liable to relapse than by any other remedy. The dose is one drachm every two hours. There is no necessity for preceding this medicine either with emetics or cathartics.

---

* *Philadelphia Med. and Phys. Journal,* February 1822.

From other sources of information respecting remedies employed in intermittent fever, we find that the efficacy of black pepper is confirmed by Dr. REIDMULLER, of Neuremberg, who has administered it to upwards of five hundred patients. The use of prussiate of iron in the same disease is announced as the subject of a treatise by Dr. FOLLICKOFFER.

From a work on the Practice of Medicine, by Dr. CRUVEILHIER,* we learn that he has experienced the happiest effects from a soft extract of the fruit of the lilac tree *(syringa vulgaris)* in intermittents. If the same benefit should result from it in the hands of others, the discovery may furnish us with an indigenous febrifuge, at a moderate price, and its use may possibly be extended, by analogy, to other disorders.

We are pleased to find that an esteemed writer on *Typhous Fever* abandons his former opinions as to one of its primary sources. We allude to Dr. ARMSTRONG:† but he thinks it not impossible to be contagious in some cases, in which the secretions are the foulest; and, as an example, mentions the nurse of patients labouring under typhus, who, having assisted one from the night-chair, became sick and faint from the odour of the evacuation from the bowels. From that time she drooped, and, a few days afterwards, had a severe attack of continued typhus.

The author had a case of intermittent fever, which in its progress put on a remittent character, and that, again, the continued, with all the most malignant signs of what is usually denominated typhous fever. It then occurred to him, for the first time, that *intermittent, remittent, and typhous fever, might be modifications of one and the same disease.*

This, in our humble opinion, is taking up the question upon the only solid basis; but can the author suppose these opinions to be new: yet he solicits communications bearing upon the subject. We would answer, that similar facts are abundantly dispersed in medical writings, which are the more to be depended upon as they are not adduced in support of any particular hypothesis. We have already stated that the intermittent fever which prevailed in the neighbourhood of Shuykill, *succeeded* the mild form of yellow fever, or bilious remittent, as it is called by the writer who describes it; and we have expressed our opinions that they both arose from the same cause, under certain modifications. During the prevalence of the bilious remittent, we are told that most of the cases were converted into intermittent, and some into typhus. Dr. Harrison observes, "the exacerbations varied from once to six times a-day. This form of fever, under proper treatment, generally terminated on the third, fourth, or fifth day; when it *changed into a regular intermittent* again." A man, on the fourth day, had all the symptoms of a confirmed typhus; on the ninth, the disease became a regular tertian ague. But, not to go to foreign authors, let us look at home. HUXHAM and SYDENHAM are in the hands of every person who can appreciate the value of such excellent observers. Let us turn to them for examples. The first that we take from our shelves

* *Medicine Pratique eclairée par l'Anatomie et la Physiologie.* Par JEAN CRUVEILHEIR, Docteur en Medecine, &c. 8vo. Paris, 1822.

† *Medical Intelligencer,* May 1822.

is Sydenham, who supplies us with all that we want, in the following extract from the beginning of his book on Epidemic Diseases.

"Ut cum præmature julio mense intermittentes autumnales ingrediuntur atque increbescunt non statim genuinum typum indunt (quod intermittentes vernis quidem solemne est,) sed continuas febres ita per omni imitantur ut nisi castigatissimo utrasque examine trutinaveris ab invicem discriminari non possint at retuso paulatim constitutionis vi jam in typum regularem migrunt."

It seems to be the invariable attribute of new medicines to perform miracles. We cannot discern any reason for believing that the *sulphate of quinine* can possess greater virtue in the cure of an intermittent than the bark in substance, from which it is prepared. Yet the thing is not impossible; and, as nothing short of experiment or personal observation can enable us to decide, we are bound to record the statements of respectable practitioners with regard to the efficacy of a remedy, however marvellous they may appear.

Dr. FALLOT, of Namur, has published three cases, which seem to prove the superiority of the quinine over the bark in substance: we have, therefore, translated them for our Intelligence department of the present month.

Of a work by Dr. PARK, on the *Pathology of Fever*,* we know nothing at present, but from a remark of a contemporary Journal, who rejoices that he maintains the *idiopathic* nature of this disorder. We would be ashamed even to apologize for our seeming neglect, if the circumstance which occasioned it had been otherwise than unavoidable: but the hurry with which we have been obliged to prepare this article, has rendered a perusal of Dr. Park's work, and some others, utterly impracticable. If, however, we are to understand, by the term *idiopathic*, that fever, in a very large proportion of cases, is not symptomatic of inflammation of some internal organs, the opinion is contrary to our experience, and, we think, sufficiently refuted by the observations of many physicians of unquestionable talent. Our readers cannot suspect us of an enthusiastic admiration of the new pathological school in Paris: nevertheless, there is much of truth in the doctrines of its founder, Dr. BROUSSAIS; and, though the oddities of the man, and his extravagant pretensions, may occasionally excite our ridicule, we cannot conceal from our view the facts which he has published on the subject of fever.

Dr. GAULTIER DE CLAUBRY† has added to the number of these facts, by a case of *Intermitting Fever*, arising from gastric irritation, which he calls inflammation, and which was speedily subdued by abstinence and "eau sucrée." We have noticed, in a preceding page, that extensive suppuration may exist in the lungs, with no other symptom than the paroxysm of an intermittent; which is sometimes produced even by common inflammation of this viscus.

---

* *The Pathology of Fever: being the Substance of the Gulstonian Lecture lately delivered at the Royal College of Physicians.* By J. R. PARK, M.D. &c. 8vo. pp. 161. London, 1822.

† *Journal Generale de Medicine.*

*Dysentery* prevailed extensively at Hebron, in the summer and autumn of 1820, and has formed the subject of a long dissertation, read before the Washington County Medical Society. The author condemns the ordinary practice by purgatives, which afford an additional irritation to the inflamed intestines, whose action is already so violent that their tender surface is exposed, and their vessels bleed. He denies, and with reason too, that they relieve pain and tenesmus, but rather aggravate them. The disease is considered by this writer to be nothing but an aggravated form of diarrhœa, and curable by opiates and astringents, as in the latter disorder.

In this epidemic, under the ordinary treatment, all the children died, except one, who had the disease very mildly. "Bleeding appeared to produce no good effect, except in some cases where there was considerable febrile excitement, and then it sometimes produced a slight remission of pain. Emetics and cathartics appeared to aggravate the disease. No difficulty was experienced in procuring an operation from cathartic medicines. They very readily passed through the alimentary canal, but frequently without procuring any increased quantity of feces; the only evidence of their operation being the appearance of the oil in the stools. Food taken into the stomach was frequently observed to pass quickly through, unchanged. Anodyne injections sometimes slightly mitigated the pain and tenesmus for a short time: other injections aggravated the symptoms, the rectum being so tender and irritable, that the introduction of any injection whatever was very painful, and the expulsory efforts of the intestines were so powerful and constant, that it was impossible to retain them, except for a very short time. Injections of ipecacuanha, so much extolled, were found ineffectual: in short, all the usual remedies failed in the cases of children.

"Discouraged with this ill success, astringents were resorted to. These alone, or combined with opium, when given in the commencement of the disease, immediately mitigated all the symptoms, and effected a speedy and permanent cure. More than twenty persons were relieved in a very short time by a preparation of vegetable astringents, without any evacuations being premised, or indeed any auxiliary medicines.

"This preparation was composed of the bark of white oak *(quercus alba)*, the roots of blackberry brier *(rubus villosus)*, and yarrow *(achilea millifolia)*. No certain proportion of each was used, but the oak and brier in the greatest quantity. For children, the ingredients were boiled in milk or milk and water, and sweetened with refined sugar; the strength and dose according to the age of the patient and urgency of the symptoms. The oak and brier were probably the only essential ingredients, and perhaps either of them might have answered the purpose alone. The brier root abounds in astringency; and the leaves of the yarrow possess a bitter, and the root a pungent aromatic quality, though by boiling this quality is materially impaired."

Dr. Campbell,* of Edinburgh, has furnished a long paper entitled

* *Edinburgh Medical Journal*, April 1822.

"Observations on the Disease *usually* termed Puerperal Fever." It contains, in our opinion, nothing but that with which we presume every practitioner must be acquainted,—the fact of its being a highly inflammatory complaint, sometimes of the abdominal, and sometimes of the pelvic, viscera, requiring the use of prompt and vigorous depletion for its cure. The cases are valuable, especially those whose fatal termination enabled the Doctor to record the appearances after death: some of these will be found in our Collectanea department. To the designation Puerperal Fever we object, and the author seems to be of our opinion by his use of the term *usually*. Some of his cases will, however, we suspect, be considered to bear such *decided* marks of inflammatory action as not to come within his qualification of it. One, for instance, twelve hours after a very easy natural labour, was seized with severe rigors, followed by pain in the hypogastric region, which darted towards the loins and ilia, and was rendered very acute by pressure. She was bled to the amount of thirty-two ounces, being two hours after the accession of rigors. She took a dose of sulphate of magnesia, and had a perfect recovery, without further treatment.

No mention is made, in the cases or observations, of the remarkable benefit resulting from the continued application of cold lotions to the belly, a remedy of incomparable value in the treatment of abdominal inflammation. It is not only useful as an adjuvant to bleeding, but can be employed in many cases where the loss of blood might be improper. We have also seen such good effects resulting from repeated injections of warm fomentations into the uterus, that we never neglect th m in practice.

The author enters at large into the consideration of the various opinions respecting the nature of this complaint, which he reduces to these seven:— 1st, that it is an inflammation of the uterus, 2dly, that it is inflammation of the abdominal linings and intestines; 3dly, that it is simply peritoneal inflammation in the puerperal state; 4thly, that it is an affection *sui generis*, and peculiar only to lying-in women; 5thly, that it is a putrid fever; 6thly, that it is a bilious fever; and, lastly, that it is a peritoneal inflammation, with typhus.

We are surprised that the author has not referred to the use of oil of turpentine in this complaint, which has been found beneficial in the hands of highly respectable practitioners. His fatal cases presented an admirable opportunity for putting it again to the test of experiment.

The same disease, if we may be allowed the expression, in a child six years of age, under a more appropriate title of *Peritoneal Inflammation*, is said, by Mr. THOMPSON,* of Whitehaven, in a recent communication, to have been remarkably relieved in a few days by oil of turpentine. The remedy was given in the dose of a small teaspoonful every two hours. Bleeding, by the application of four leeches only to the abdomen, had been previously employed; which was afterwards succeeded by a blister to the same part.

An instructive review in the Edinburgh Journal has made us ac-

* *London Med. Repository*, April 1822.

quainted with two treatises on the *Radesyge*, or *northern leprosy*.* We are, very fortunately, not liable to the visitations of this hideous malady; but, viewing it in connexion with cutaneous and endemic disease in general, the subject is highly important. The article in question will prove a valuable object of reference to the future inquirer, not only from its intrinsic merit, but as directing to other sources for details, which it would have been impossible to compress within the ordinary limits of similar productions.

The disease has been described, by various writers, as allied to *scurvy, lues,* or *Arabian leprosy;* and these discrepancies are considered, in some degree, to be evidence against the justice of either opinion. The most material part of this inquiry relates to the identity of radesyge and Arabian leprosy, which is unhesitatingly assumed to be the fact by the most eminent physicians. The reviewer, however, decides in the negative, for the following reasons:

"1st. There is no proof that the Arabian, or true tubercular leprosy, as it appears in Madeira, the West Indies, &c. is at all contagious; for, in every stage of it, the lepers associate, in the closest and most unrestrained manner, with healthy persons, who never, on that account, become infected. The opinions of the physicians of the middle ages on this point can scarcely be trusted with much confidence. The radesyge, on the other hand, is stated, on the most creditable authorities, to be highly contagious; and the utmost precautions are requisite to prevent the disease from being diffused, when it has once made its appearance in a district.

"2. The observations of Dr. Adams show that the disposition to leprosy, if not the actual disease, is hereditary. We cannot discover any thing of this nature in the history or formation of the radesyge, which seems to be so little under the control of the hereditary influence, that it has entirely disappeared in some situations, where the external causes have either become less powerful, or have entirely ceased to operate.

"3. The leprosy and radesyge differ very much in their progress, and in the morbid changes which take place; for the former is a tedious and chronic disease, continuing many years stationary after it has fully developed itself, in the form of tubercles, and without, in most cases, assuming the ulcerative process; while the radesyge proceeds with considerable rapidity from the tubercular to the ulcerative stage: and here particularly it resembles, and is liable to be mistaken for, a syphiloid malady.

"4. The circumstances which we have now enumerated as demonstrating a difference between the radesyge and the tubercular disease of the East, are by no means unimportant in establishing a line of demarkation; but they are very immaterial when contrasted with the effects produced on the genital system by the Arabian distemper. In

* *Morbus quem Radesyge vocant, quænam sit, quanamque ratione e Scandinavia tollendus?* Commentatio, Auctore FREDERICO HOLST, Medicinæ Doctore. 8vo. pp. 157. Christianiæ, 1817.

*Ueber die aussatzartige Krankheit Holsteins, allgemein daselbst die Marschkrankheit genannt.* Von LUDWIG AUGUST STRUVE, Dr. &c. 206 seit in 8vo. 1820.

all the cases, both in males and females, which Dr. Adams examined, he found that the occurrence of elephantiasis uniformly destroyed the characteristic distinctions of sex, if after puberty, and prevented their development, if before. Not only were the functions, as it were, obliterated, but the texture of the organs seemed to be much altered and impaired. Nothing of the kind seems to take place in the radesyge; and, if the functions of these parts are affected, it is evidently in consequence of the injury done to the constitution at large. We might add, though purely as an occasional occurrence, that the cornea is often injured, and the sight destroyed in the unfortunate lepers; while nothing similar is remarked in the northern disease.

"Lastly. We may here remark, as connected with this part of the subject, the tendency in the radesyge to wear itself out, as it were, or, in other words, the possibility of its being cured in particular circumstances."

Not having ourselves seen the disease, nor having had the means of tracing the steps of the learned reviewer in his researches upon this occasion, it would be presumptuous in us to give an opinion. We may, however, be permitted to make a suggestion, the truth of which will be immediately obvious, that the same disease may vary considerably, under the influence of local or other circumstances. It is not impossible that, were the lepra of Madeira transported to the inhospitable climes of Scandinavia, and made to partake of the diet of the lower orders of its inhabitants, his tubercles would take on the ulcerative action, and thus become contagious. We mention this because we do not think the contagious nature of the disease, before ulceration, is clearly made out. The writer says, the radesyge is most readily and certainly communicated by the matter of the sores which are generated in its progressive advancement. We object, also, to the formation of sweeping conclusions, in the absence of information on some points which are of great importance to be known. It is admitted by the writer that the evidence is extremely defective.

Dr. CRANE, of Boston, relates a case of *Bulimia*,* in which the patient regularly took, at each meal, three or four pounds of meat, besides bread and vegetables. Vomiting generally supervened after eating. The food was ejected unchanged, and accompanied with a large quantity of glairy and mucous-like matter, decidedly acid to the taste. Schirrous ffection of the pylorus was suspected by the practitioners formerly consulted, and corresponding treatment recommended, without success. During a severe attack of fever, all desire for food left her, but returned with her convalescence. The morbid appetite was removed by persevering abstinence, as far as we can collect from the confusion which pervades this part of the account.

An American Journal contains an article on the *Humoral Pathology*, a favourite matter of speculation with us. It has long been the fashion to ridicule this exploded doctrine; so much, indeed,

* *London Medical Repository*, April 1822.

that, to be its advocate, even in a limited degree, is considered by many to be the extreme of absurdity: we are not ashamed, however, to express our belief that many maladies have their origin in a deteriorated condition of the blood. Neither the solids or the fluids can, indeed, be regarded as the exclusive seats of disease: we believe that each of them is susceptible of morbid changes and impressions, capable of involving the general system.

A well-written essay, in illustration of the truth of these opinions, has been published by Dr. EBERLE.*

Evidence might be collected on every side to prove the capability of the blood to become the receptacle of morbid humours, and, in consequence, to engender disease of various kinds. The writer, accordingly, has not neglected to adduce so much as is necessary to support the cause he maintains. We may dispense with any reference to, or quotation from, these authorities, as sufficient evidence will be found in our Critical Analysis of Dr. GASPARD's *Physiological Memoir on Purulent and Putrid Diseases,* which disorders the author produced in dogs by the injection of putrid animal and vegetable matter into their veins. It will be particularly remarked, on the reading of the experiments made with putrid matter, that morbid appearances were found in the animals subjected to them, similar to those which are seen in dysentery and putrid fevers, generally admitted to arise from an operation, in some way, of putrid miasma on the animal economy. So, in a series of experiments formerly made by Dr. Gaspard, to determine the effects of acetate of lead, it was found that, when injected into the veins, symptoms analogous to the painter's cholic were produced; and the intestines of the animals, on dissection, were found to be considerably inflamed. On comparing the symptoms of those in whose veins the poison was introduced, with those of dogs who contracted the disease in consequence of being kept in a manufactory of this salt, they were found precisely the same.

*Sympathy,* as opposed to *Humoralism,* has found advocates in Drs. GELCEN, of Paris,† and Dr. CALDWELL, of the United States.‡ The zeal and style of the latter are truly remarkable: his opinions are contained in a long letter to Dr. CHAPMAN, his friend and ally in this war against the Philistines. Although, says Dr. Caldwell, "not perhaps justified in saying that, in behalf of the sympathetic school, we have maintained the conflict entirely alone, it is certainly true that, as teachers and disputants, we have, for many years, held our stations in the front rank of battle. Nor, while supported by an ally so able in council, and so dextrous in combat, as you are, does any apparent augmentation of the forces of our opponents awaken in me the slightest apprehension or concern. However few in numbers they may originally be, the advocates of truth, and the faithful interpreters of nature, cannot fail, in the end, to triumph over legions entangled in error. For a time, indeed, the struggle may seem desperate and the issue doubtful. The "million" may even exult in the anticipation of a certain, an easy,

---

* *American Medical Recorder*, January 1822.

† *Journale Complementaire,* Mars 1822.

‡ *Philadelphia Med. and Phys. Journal,* January 1822.

and a speedy victory. Like the small but invincible band of BYRON's Conrad, the advocates of sound principles may be considered

"Hemm'd in—cut off—cleft down—and trampled o'er."

But the fancy is illusive, and the hope a bubble. Let them be true to their cause and to themselves,—let their resolution be manly, and their perseverance inflexible,—let them, in the language of the same illustrious favourite of the Muses,

"——each strike singly, silently, and home;"

and, instead of falling themselves in the deadly conflict, it will be their antagonists that must

"——sink outwearied rather than o'ercome."

We are quite aware that many of the phenomena of animal life cannot be explained without calling in sympathy to our aid. On the other hand, much which is ascribed to sympathy is owing to the direct absorption of matter into the blood. Our space will not permit us to follow these authors by an examination of all their tenets; but one or two we shall very briefly notice. "Arsenic, (observes Dr. Chapman,) spider's web, and Peruvian bark, act directly on the stomach only, in the removal of intermittent fever. On the system at large, they operate exclusively through the medium of sympathy:" this we beg to deny.

Some of the notions of Dr. GELCEN appear to us no less objectionable. For example, he says, "I knew a young girl in whom the menstrual discharge was suppressed by spasmodic tension. She was bled in the foot. The vein was no sooner opened than the menstrual evacuation returned." What does this prove?—Again. "There are pulmonary consumptions whose essential cause is a radical weakness of the pulmonary organs, on account of which they are gorged by matters that transform themselves into tubercles. It is in these cases that the treatment advised by REID produces salutary effects: this consists in fortifying the lungs by acting on the stomach with small and repeated doses of ipecacuanha." In the latter case, we suspect the author is not only wrong in his conclusion, but as to his fact. Is it tubercular consumption that is cured by ipecacuanha? And, admitting this hypothesis to be true, where is the proof that the remedy operates on the lungs by sympathy, instead of absorption into the circulation?

When Dr. Caldwell expresses himself with such entire confidence relative to the *modus operandi* of bark on the stomach being ascribable to sympathy, or, in other words, to nervous agency, we think he should have explained his mode of accounting for those facts which are related by Mr. BRODIE, to prove that the poisonous substance, *woorara*, affects the system only *after having entered the circulation*: for instance, he exposed the sciatic nerve of a rabbit in the upper and posterior part of the thigh, and passed under it a tape half an inch wide. He then made a wound in the leg, and having introduced into it some of the woorara mixed with water, he tied the tape moderately tight on the fore part of the thigh. He thus interrupted the communication between the wounds and the other parts of the body by means of the vessels, while that by the nerve still remained. After the ligature was tightened, he applied the woorara a second time in another part of the leg. The rabbit was not at all affected, and at the end of an hour he removed the ligature. Being engaged in some other pursuit, he did not watch the animal so closely as he should otherwise have done; but, twenty minutes after the ligature was removed, he found him lying on one side, motionless and insensible, evidently under the influence of the poison.

The *modus operandi* of medicines forms an important branch of this inquiry; for, if they are absorbed into the circulation, morbific matter may find an entrance in the same manner; and that such is the fact, we think demonstrable. Can Dr. Caldwell excite vomiting by applying emetic tartar to a naked nerve, so as to preclude the possibility of absorption? No. Yet, taken into the stomach, or injected into the veins, the effect will be certainly produced.

We are very reluctantly compelled to abridge our remarks, from a fear of exceeding our limits: we shall, therefore, confine ourselves to one quotation from Dr. Eberle:

"It is alledged, by the advocates of the sympathetic doctrine, that chyle is always essentially of the same nature, however various the substances from which it may be formed. The converse of this opinion is, however, proved by a number of facts, too powerful for mere speculative reasoning to controvert. If the nature of the food do not modify the chyle, and consequently the blood, how is it that the secretions, as well as the general state of health, are so much under the influence of our diet? Dr. Stark, many years ago, restricted persons to a diet composed entirely of sugar and water. The persons thus fed were affected, in the course of some weeks, with scorbutic symptoms; the gums became spongy and covered with ulcerous spots, and sores broke out on different parts of the body. Latter experiments confirm those of Stark. Indeed, it is now well ascertained that symptoms of scurvy may be produced, by confining persons either to an exclusive animal or vegetable diet. That the scorbutic symptoms thus induced are mainly dependent on a morbid condition of the blood, is not improbable, both from the altered appearance of the blood, which is thick, viscid, and black, and from the disease being relieved by a recurrence to a mixed or opposite diet. Lind, Blane, and Millman, regard scurvy as a disease of debility, having its primary seat in weakness of the digestive organs; and as owing its origin to a defective, rather than a vitiated, nourishment. Debility of the digestive organs is certainly among the earliest symptoms of disease. The stomach cannot long bear either a vitiated or exclusive diet, without suffering in its powers. But, as we have no example of scorbutic symptoms arising from mere indigestion or debility of the stomach, we are forced to admit the presence of some other cause; and, as we have unquestionable marks of a morbid alteration in the state of the blood, we have a right to conclude that this circumstance contributes to the production of the peculiar symptoms of the disease in question. That the appearances of the blood, however, are much influenced by the state of the digestive organs, has been particularly noticed by medical writers. Whatever the character of our food be, if the chyle be imperfectly elaborated, in consequence of a weakness of the digestive organs, considerable changes take place in the blood from its ordinary or healthy appearance. 'In those who have long laboured under indigestion,' says a late writer, 'the blood is sensibly altered in some of its properties. The proportion, both of red globules and lymph, is less than in health.'

"Independently, however, of the effects of indigestion, it is evident, from a variety of facts, that the nature of the food itself has a very considerable influence both upon the blood and the secretions. Animals that live wholly upon flesh have a urine which consists almost entirely of uric acid: in graminivorous animals, on the contrary, the urine contains no lithic acid at all. And, in man, it appears that the urinary deposits are constantly modified according to the different kinds of nourishment and drink taken. How are these effects produced? Is it by the action of the food on the nerves of the stomach and bowels, and thence propagating a sympathetic action to the secretory vessels of the kidneys, enabling them thus to convert a larger proportion of the ordinary elements of the blood into the substances in question? Or does the chyle, which is formed from certain kinds of food, convey a larger portion of the elements of such substances into the circulation, and furnish a more abundant stock of materials to the secretory glands? This latter opinion derives strength from the observation of Magendie, that the lithic acid is increased by an animal diet, in consequence of its furnishing the azote, which is essential to the production of this acid.

"We have strong evidence, also, of the influence of diet, both upon the physical and moral constitution of man, by adverting to the character and diseases of such people as, either from habit, necessity, or superstition, confine themselves almost entirely to one particular kind of food. The Norwegians, Siberians, and Laplanders, who live almost exclusively on fish, are said to be pusillanimous, and subject to scorbutic and other cutaneous diseases. 'Nul doute qu'll n'en (la nouriture de poisson) résulte l'introduction de principes âcres et nuisibles dans nos corps. Que delà naissent des dispositions au scorbut, des affections cutanées rebelles, des gales des dartres, dans les climats froids.' It is stated by other writers, that ichthyophagists become languid, feeble, and overburthened with soft and oily fat. Such persons are also much subject to cutaneous diseases. The inhabitants of the sea-coast, those of Lower Brittany, of Biscay, and along the borders of the Baltic, are very much affected with the itch, scurvy, and tetter."

Since the humoral pathology has been degraded from the schools, the histories of *Conversion of Disease,* which abound in the writings of the older physicians, seem entirely to have lost their influence upon the practice of the moderns. Although attempts have been made, at various times, by gentlemen of high consideration, to rescue from oblivion these important facts, their efforts have been attended with but transitory benefit. Among the valuable contributors upon this subject may be reckoned the late Dr. FERRIAR, of Manchester. Another has recently appeared in the United States, who supplies an excellent essay on *Metastasis:* we allude to Dr. HARRIS, of Philadelphia.*

---

* *American Medical Recorder.*

Some of the facts which he has collected, although not without parallel, are so curious, that we shall transcribe them, having no doubt of their being acceptable to the bulk of our readers.

LECLERC informs us, that a lady was affected with a tumor of the breast, which had been thought cancerous, and for which many remedies had been employed without advantage. At length she lost all hope, and abandoned the case to nature. After a time she was affected with a tumor in the leg, which rapidly increased in size and suppuration. The discharge being very abundant, the cancer in the breast diminished, and finally disappeared. A physician of celebrity advised her never to suffer the ulcer, which had produced so happy an effect, to cicatrize; but, becoming tired of its inconvenience, and feeling otherwise in robust health, she urged her surgeon to heal it. He yielded to her importunities, cured the leg, and was rewarded by a re-appearance of the cancerous tumor of the breast. Suppuration was again established in the leg, and maintained there by the application of caustic, which produced a complete cure of the original disease.

Dr. PARRISH endeavoured to induce a metastasis in a consumptive boy, by the introduction of vaccine matter into the glands of the neck. By this practice he succeeded in establishing an external scrofulous ulcer, which happily resulted in the cure of his patient of a form of disease which doubtless would have destroyed him in the course of a few months.

A patient inherited pulmonary consumption; and, after the disease had progressed so far that her life was despaired of, the Doctor, with great judgment, irritated the glands of the axilla, promoted suppuration by emollient poultices, and thus brought about a scrofulous ulcer, which completely cured her of her pulmonic symptoms. When he last heard from her, she was in excellent health.

There are periodical evacuations, that result either from an hereditary predisposition, or are developed by incidental circumstances, but which, from long continuance, are so familiarized with our organs, that they become at length new functions indispensable to health. These evacuations vary to infinity. Sometimes they are habitual and abundant sweats; at other times, they consist in either hemorrhages or purulent discharges by the natural emunctories.

If such complaints are known to be of some duration, or are suspected to be inherited, the only service required of the physician, under such circumstances, is to prevent a metastasis. The ill effects resulting from suddenly arresting these drains, might be illustrated by a thousand cases drawn from the best authorities.

RAYMOND mentions that a nun had been long affected with a purulent discharge from her eye-lids, which, at the age of twenty-two, yielded to copious and fetid sweats from her legs and feet. While she submitted to this inconvenience, her eyes continued strong and healthy. At length, however, the sweats so increased in quantity and fetor, particularly during the summer season, as not only to be offensive to herself, but to inconvenience those with whom she associated. In order to remove a complaint in every way so disagreeable, she bathed her extremities in astringent washes, which arrested the cutaneous discharge, but brought on an obstinate epilepsy, with which she was affected, at intervals, for three years. In consequence of one of those changes of predisposition, about which we know so little, she was relieved of her epileptic symptoms, and became affected with scrofula in the neck and axilla. For want of proper management, the disease was translated to the lungs, of which she shortly afterwards died.

Reydellet records several cases of nearly the same character as the above, and in which the suppression of the perspiration was attended with equally unhappy consequences.

In March last, the author was called to visit a lady affected with severe pain in the back and head, with great depression of spirits, and occasional symptoms of hysteria. He had her under treatment nearly a month, without being able to lessen, in any degree, the violence of her symptoms. About this time she informed him, that, since her indisposition, she had gotten rid of an old companion, (as she termed it,) which was extremely offensive both to herself and friends. Upon inquiry, he now found that she had originally laboured under a scrofulous tumor of the groin, which, about three years since, had yielded to copious and fetid sweats of her feet. With this inconvenience she enjoyed perfect health, until she had received her recent cold, which she supposed was obtained by getting her feet wet. Recollecting the case of Raymond, he was convinced that the proper indication of cure consisted in inviting the return of the perspiration to her legs and feet. For this purpose, pediluvia, the vapour bath, and emollient poultices, were used, with the completest success. Her fetid sweats, health, and good spirits, returned together.

Almost every attempt to cure hemorrhages of long continuance is attended with unhappy consequences. There are few physicians in extensive practice who have not witnessed examples of this kind. Dyspepsia, hepatitis, consumption, epilepsy, or apoplexy, is produced by metastasis; and which can be permanently cured in no other way than by inviting a return of the hemorrhoidal disease.

No disease is so subject to metastasis as gout. To cite ordinary cases of this kind would be useless, as they are known to every practitioner. Ferriar records some curious cases of gouty consumption, which merit attention; and which, in most instances, he cured by the exhibition of cordials, and thereby repelled the disease to the extremities.

Venereal metastasis is not unfrequent. Hunter, Bell, Astruc, and others, have published many interesting cases of this nature. Reydellet states that he had a young man under his care affected with gonorrhœa, who, under the ordinary treatment, appeared to be doing well. Being obliged to expose himself in cold damp weather, the gonorrhœa suddenly ceased, and an inflammation of the right eye supervened. On this metastatic affection he exercised all his skill, but without being able to even ameliorate its violence. At length, however, the discharge from the urethra spontaneously returned, when the pain and inflammation of the eye immediately subsided. Dr. Plenk was consulted in a case of ophthalmia of this character, for which he advised his patient to contract a new gonorrhœa. The patient expressed no reluctance to follow the advice, and was compensated by a rapid and complete cure.

Cutaneous affections of long continuance should be treated with great caution. Ferriar notices "a remarkable case in which epileptic fits were produced by the retrocession of the itch, in consequence of some external application. In this case, the epilepsy resisted all the usual methods, and was only cured by reproducing the itch." Mania and hypochondriasis have been produced by the same causes, and cured by the same means.

Puerperal fever is, doubtlessly, a metastatic affection, as it is generally attended with suppression of the lochia. By taking this view of the disease, the powers of turpentine as a remedial agent may be readily explained. It is known to act with great force on the pelvic viscera, by which means it repels the metastasis to the organ primitively affected, and thus produces a return of the lochia.

These cases, it will be observed, are collected from other authors, and are mostly of old date; but two very similar ones have occurred within the period included in this Retrospect. The first was that of a woman,* subject to frequent and copious pustular eruptions in the head; a few months after the drying-up of these, she experienced painful chilliness and lassitude, which was soon followed by acute pain under the breast, dyspnœa, dry cough, pains of the head, with bitter taste in the mouth, and fever. On the fifth day of the disease, the respiration had become natural, and the pains in the head and breast had left her. The treatment pursued in the interval was bleeding, vomiting, and purging, which brought away much bilious matter; and a blister was applied to the right arm. From the fifth to the seventh, the prostration of strength was great, the belly was tense, accompanied by constant liquid stools, which were checked by wine, enemata of bark, and opiates; spirits of wine and camphor were also applied to the sphincter ani. On the cessation of the purging, she had two or three bilious vomitings. From the eighth to the tenth, the vomitings were more frequent, with hiccup, and burnings in the throat, excited by the passage of wine, which increased to a degree almost amounting to erosion; the tongue was covered with a thick white mucus. Between this period and the thirteenth, membraniform flakes were voided by stool; the hiccup first diminished, and then ceased altogether, with a spontaneous bilious stool. Convalescence now began to be established; when, on the twentieth, the pulse on the left side became weaker and difficult to be felt. Violent pain came on through the whole of the left arm, with coldness and torpor of the extremities. The subclavian ceased to pulsate, but the carotid preserved its natural action. The arm was immersed in hot water. Bottles filled with it were applied to the hand and fore-arm. Opiates were given; and, on the twenty-first, two grains of the extract strychnos, at two, at five, and at eight o'clock. On the twenty-second, pain was experienced in various parts of the arm, on pressure. Some of the fingers were benumbed, others were painful at their apices; the urine was of a lemon colour, white and turbid at the bottom of the chamber-pot, with lateritious sediment. Eight grains of the extract were taken during the day; nourishing and stimulating diet

* *Journale Generale de Medecine*, January 1822.

continued; with a blister to the levatores scapulæ. Twenty-third day, the course of the biceps and pronator teres muscles was still painful on pressure, but the sensibility had returned in the fingers. Redness and perspiration spontaneously occurred in the right shoulder. On the twenty-fourth, evident pulsations were felt in the radial artery; swelling took place, with pain, in the bend of the arm. On the twenty-seventh, the swelling was diminished, but the pain was unabated; the jaws, also, had become painful. Twenty-eighth day, the blisters were healing, and the pains were more intense. At length, boils appeared on the right shoulder, with diminution and final disappearance of the symptoms.

We are indebted to Dr. STAUGHTON* for the record of the second. The disease was an abscess, which occupied the space between the lower jaw and sternum in a child, seven years old. He had slight paralysis of his inferior extremities, with tension of the skin and pain over the right knee. The abscess burst with relief of the symptoms; but the orifice closed, and he discharged from the rectum four or five ounces of matter, resembling that which came from his neck.

An instance of recovery from *Meningeal Inflammation*, in a little girl eight years of age, has been recorded by Dr. FALLOT, under the title of *Hydrocephalus*. Thanks, however, to any thing but the vigour of the practice employed, it did not, in our opinion, run into the stage of effusion, which could alone entitle it to the latter denomination. After seven days of indisposition and complaint of pain in the occipital region, the child had constant inclination to vomit, flushed face, intolerance of light; red dry tongue; burning skin; small, hard, quick pulse; lancinating pains in the head, especially over the orbit, causing the child to utter piercing cries. The surface of the body was soon covered with a purplish eruption, which desquamated on the third day of its appearance. On the fifth day from the accession of these marked symptoms, the pulse had increased in velocity; the face was drawn, and the eyes were incapable of bearing the least degree of light. The author now, for the first time, suspected hydrocephalus, and determined to act *vigorously*, by enveloping the feet in sinapisms, giving a grain of calomel every three hours, with an antimonial solution, and covering the head with ice. This is all very well; but why not bleed? On the commencement of febrile symptoms, leeches were applied, but by no means in quantity sufficient to subdue a disease so generally fatal. For the space of a week she continued nearly the same; when we have the following account, to which we beg the attention of the reader, in connexion with the opinion we have expressed, that effusion had not taken place.

The breathing was oppressed, the anxiety extreme; the patient attempting to raise herself, but wanting the power. When elevated by others, the head fell to one side or the other. The heat of the skin was burning and unequal, with somnolency, interrupted by startings and crying out. About three o'clock in the morning, convulsive move-

---

* *American Medical Recorder*, January 1822.

ments took place over the whole body. At day-light, she squinted horribly; the right pupil being dilated, and scarcely sensible to a lighted candle. The intellects, however, appeared sound when the patient was roused from her slumbers. The features were much altered; the skin burning; the pulse small and irregular; with grindings of the teeth and piercing cries. In two days the convulsive movements ceased, but left an inability to move the left arm and leg.

The latter of these symptoms, with dilation of the pupil and squinting, to say nothing of the convulsions, are so generally considered to be the marks of pressure on some part of the brain, that we have thought it our duty to bring the subject under consideration. No error can be more fraught with danger than that which ascribes to effusion the effects of inflammation of a vital part. To say nothing of our personal experience, several cases are related in the treatise on Arachnitis, by Drs. PARENT and DUCHATELET, which prove that the symptoms above mentioned have existed during life, although no signs of effusion were discoverable on dissection.

Another case, in which coma was evidently symptomatic of inflammation, is related by M. GENDRIN.* On the 20th of June, a man was attacked by fever, pain in the limbs, nausea, and excessive head-ach. By leeches, vomiting, and purging, the head-ach diminished; but slight delirium came on, which however was of short duration. On the fifth day, the delirium became continued and complete; the jaws were spasmodically contracted; the tongue was dry and black, and the teeth encrusted with a black coat. On the tenth day, he was in a state of coma, from which he was roused with difficulty. His conjunctivæ were turgid; his jaws firmly fixed, and almost immovable. He had subsultus tendinum, with tetanic rigidity of the limbs, appearing and disappearing on each side alternately, and sometimes in both together. The thorax and abdomen were in their natural state. Six leeches were applied to each temple, and sinapisms to the legs, without benefit: he was afterwards bled, and cold affusions, among other treatment, were employed to the head, &c.; under which the patient recovered.

We think, also, we can discover evidence of the same fact in a case of *Hydrocephalus* in a child, which recently occurred to Mr. BRICHETEAU.† Stupor and dilated pupil (among symptoms which we consider more particularly to belong to inflamed brain,) were removed by bleeding with leeches. These symptoms returned; but, again, in the course of the disease, all the cerebral symptoms disappeared,—a result which, we suspect, would not have been witnessed had effusion taken place. As these facts would be better appreciated in connexion with the other particulars of the state of the child, we subjoin the most material of them, nearly in the words of the narrator.

On the ninth day after general indisposition, the child was in a state of stupor; the eye-lids were closed, and the pupils dilated and immovable; the look was fixed, the eyes dim, and covered with a sort of mucous pellicle. The tongue was yellowish at its base, dry and red at its

* *Journale Generale de Medecine*, Fev. 1822.

† *Journal Complementaire*, Fev. 1822.

point; the head was heavy, and turned backwards; the skin hot, with acute fever. On the tenth, to these symptoms was superadded an almost continual rotatory motion of the head. Sinapisms were applied to the feet, and leeches to the neck, which bled very copiously, and produced syncope. On the following day, the stupor disappeared, and the pupils became movable; but in the evening the symptoms recurred. On the thirteenth day, the stupor was interrupted from time to time by piercing cries: the rotation of the head continued. On the fifteenth and sixteenth, the child had a continual purging of greenish matter. On the seventeenth, *a tumor appeared behind the angle of the lower jaw*, and on the following day the amendment was very remarkable: the stupor disappeared, the pupils resumed their functions, and the fecal matter became yellow. The child took food, and was taken out into the air; but in the night the diarrhœa increased. Until the twenty-third day of the disease, the cerebral symptoms were absent; but the child refused food, the diarrhœa continued, and the hands, feet, and legs became anasarcous. On the twenty-third the stupor re-appeared; the eyes were sunk, and covered with mucous pellicles. The tumor of the neck became inflamed; caustic was applied to it; and, on the following day, four spoonfuls of matter were extracted. On the twenty-fifth and twenty-sixth day, the face became cadaverous, and the tongue was covered with a dark coat: the cries were piercing and constant; the anasarca increased; and on the twenty-seventh it died.—Water was found in the ventricles on dissection.

A fact, which bears upon this subject, is recorded by Dr. MACLEOD,* in his Report of the Diseases of London. In two children, the symptoms supposed to characterize effusion had come on some days before death. In one, the ventricles contained about three ounces of fluid; in the other, they contained none. In the latter, a transparent gelatinous matter, like white currant jelly, was effused underneath the arachnoid membrane, particularly in the hollows formed between the convolutions. This is not the first example, the Doctor observes, of a disease running the exact course of water in the head, in which none was found.

The suggestion of Sir G. BLANE to employ pressure in cases of *Hydrocephalus Externus*, has been successfully put in practice by Dr. GIRDLESTONE, of Yarmouth; the particulars of which will be found in our Number for March last. We are not, however, sanguine in our expectations of frequent recoveries under this treatment. It may be well to put into contrast, and to reconcile, if possible, with the facts related by Dr. Girdlestone, the judicious remarks made by Dr. JEFFERY, the professor of anatomy in the university of Glasgow, on the dissection of a case of this kind.† The disease had existed in a child of about nine years of age, for the space of six years. Four quarts of fluid were found within the ventricles. The writer observes, that, while the sutures of the cranium permit the brain to expand, little or no inconvenience is experienced; but, when they become firmly adherent, the pressure upon the brain is insupportable.

* *London Medical and Physical Journal*, April 1822.
† *London Medical Repository*, April 1822.

We may here notice, without any disposition to detract from the merit of Sir Gilbert Blane, by an insinuation of plagiarism, that the employment of pressure in cases of hydrocephalus is not new. RIVERIUS relates that a surgeon of Montpellier cured a congenital hydrocephalus in this manner; and we have heard of other instances, though we cannot immediately call them to our recollection. An instance of the cure of *Ascites* by compression has also been recorded by Dr. HUSSON,* the physician of the Hotel Dieu in Paris. After the usual remedies were employed without permanent success, the patient was tapped, but the abdomen began to fill again; in consequence of which a bandage was kept tightly applied round the belly, and in seventeen days she was dismissed cured. It was remarked that the urinary secretion increased under the influence of this treatment.

We should be glad to see the practice employed in *Hydrocele.* To those who may be disposed to give it a trial, we may suggest that it might be convenient first to evacuate the contents of the tumor by simple puncture, and then to bind up the testicle with adhesive straps.

Most of our readers must be acquainted with the cases of Mr. BAMPFIELD, intended to show the identity of *Variola* and *Varicella.* We are not, however, satisfied with the conclusions of the writer; conceiving that the cases supposed to be varicella are small-pox modified by the previous vaccination of both the patients. Dr. REED,† of Kilmarnock, however, on the authority of several cases which he has published, is also of opinion that small-pox, the varioloid disease, and varicella, will be found to be identical. "We have in proof, decidedly severe small-pox giving origin to this disease, often trifling in its nature, and always short in its duration; and from this, again, has been reproduced severe confluent small-pox; vaccination appearing the only modifying power, and affording the sole reason why the disease in one was severe, and in the other slight. The inference, therefore, I conceive legitimate, that diseases producing and reproducing each other must be the same."

If the latter, indeed, be proved, the case is made out; but are not the supposed cases of varicella small-pox modified by vaccination? and is there any well-authenticated instance of *unequivocal* varicella having produced small-pox? Varicella was known as one of the commonest of the diseases of children, long before vaccination was introduced. How is it that it was never, until recently, suspected to possess this power of generating variola?

The important document of this author supplies additional evidence of the fact, that vaccination, in a vast number of cases, is not a *complete* security against small-pox: that, where the latter prevails epidemically, in the midst of patients who have been vaccinated, many of the latter are susceptible of variola, in the modified form in which it so strongly resembles varicella.

In Kilmaurs, sixty cases occurred. One-sixth confessedly had not

* *Annuaire Medico Chirurgicale.*

† *Edinburgh Medical Journal,* April 1822.

been vaccinated, and of these one died; among the remainder, no deaths took place. It should be here noted, that a girl, of twelve years old, who formerly had small-pox without inoculation, now fell a victim to a second attack.

In Kilmarnock, fifty cases occurred. One-fifth were unprotected by vaccination, and of this number two died: no deaths took place when vaccination was performed.

A varioloid epidemic occurred at Strathaven: one hundred cases occurred; one-eighth were unprotected, and of these, three died: no death among the vaccinated.

A strong case is given by the author of the protecting power of the vaccine inoculation, performed on a nurse during her attendance on a case of small-pox.—"A young man passed through a room, wherein laid a child covered with small-pox; never having himself had the disease, nor having been vaccinated, owing to the obstinate prejudices of his father, he felt an involuntary shuddering at the sight of a disease, of which, till then, he had not formed an idea. A day or two after his return he was seized with fever and pains, which I believed those of rheumatism, till, by dint of interrogation, the foregoing account was elicited. The eruption put an end to all doubts on the subject. So soon as this point was settled, his nurse, a fine-looking woman of twenty-five years of age, of a clear complexion, lately married, and pregnant, mentioned that she had never been inoculated, nor had undergone the disease in any form. She was immediately vaccinated in both arms. The operation succeeded well; and, although she was about the patient constantly during several weeks, in a situation peculiarly unpleasant, surrounded by an atmosphere loaded with the sulphurous putrid effluvia peculiar to small-pox, she escaped with perfect impunity, and became, four months after, the mother of two healthy children."

So slight are the accidents by which the thread of life may be severed, that we ought not to be surprised that even *vaccine inoculation* may occasionally produce fatal consequences: such has been the case in a child under the care of Dr. LUCAS, of Hatfield.* An areola of inflammation, of the usual extent and appearance, surrounded the vesicles; and in the evening the child was taken rather suddenly ill, with fever and uneasiness, with a slight expression of tenesmus. The next morning the inflammation extended from the shoulder to the wrist, considerable fever being present. Cold rose-water was applied, and the child was purged well with calomel, antimonial powder, and rhubarb. On the following day, the inflammation had spread over the left breast, top of the shoulder, and shoulder-blade; the pulse very rapid, about fifteen strokes in five seconds; and the child lay in a quiet, half comatose state, but crying much when disturbed. The purgative was repeated with effect; and a warm lotion applied of the liq. ammon. acet. On the twelfth day, the inflammation having somewhat abated, the lotion was laid aside; but, increasing again considerably in extent and intensity, on the thirteenth it was resumed. From this time the

---

* *London Medical Repository*, June 1822.

inflammation continued to extend itself, gradually receding from the parts first attacked, until it successively covered the whole body and limbs, except the feet and upper parts of the face and head; a degree of œdema following it, particularly affecting the scrotum and lower limbs. The bowels were kept open by the magnes. sulph.; from six to twelve daily evacuations being procured through the whole course of the disease. These were greenish, curdly, and somewhat slimy; but not differing materially, as the mother said, from the usual evacuations of the child. The symptoms continued in this state until the seventeenth day, when sickness came on, with some fullness of the belly; and on the morning of the eighteenth day the child died, with every appearance of internal mortification, the abdomen being very tumid, with livid colour of the integuments in patches; and a great quantity of thin, dark-coloured fluid being dejected by vomiting through the mouth and nostrils. The body was not opened; but there is reason to believe that the same inflammation, affecting the mucous membrane of the bowels, was the immediate cause of the fatal event, as the child had outlived the external inflammation; which, however, did not entirely disappear until within twelve hours of its death.

Experiments have been made by a Dr. RANKEN,* of the Bengal Medical Establishment, upon dogs bitten by the *Cobra de Capello*, to determine the remedies proper to be employed in similar cases. The style of the paper, and the experiments themselves, are very juvenile; nor have the latter been performed to an extent, and with an accuracy, to warrant the conclusions drawn from them by the author. The author sets out by stating, that he has seen about twenty persons who had been bitten, " and were *evidently affected*, yet recovered on taking the volatile alkali: but in none was the kind of serpent satisfactorily ascertained. He never saw a person cured whom he knew to be bitten by the hooded snake." Now, having the evidence of twenty cases of recovery from the volative alkali before his eyes, it seems a little surprising that he should have commenced his experiments with another medicine; yet we find that he began with the oil of turpentine. The author then illustrates the principles which influenced his practice. " Having observed the venom to produce at first violent excitement, which soon causes extreme depression, I had formed an opinion, after Mr. Williams, that the cure must consist in the counteraction of some powerful stimulant. Further experience has tended to confirm this doctrine; and, in consequence, the remedies employed in these experiments are all of that class. On the same principle, too, I accounted for the inefficacy of the alkali as administered by Fontana, for whose accuracy and candour I entertain much respect. He exhibited it as soon as the animals were bitten: in other words, a strong stimulus acting simultaneously, instead of counteracting the poison, was made to assist it in exhausting the powers of life; and, having seen men recover when the medicine was given after they had been visibly infected, I conceived that the curative process, or counteraction, ought not to commence till then. The three first dogs are therefore treated in conformity with this theory." The

* *Edinburgh Medical Journal*, April 1822.

puerility of the author is abundantly shown by these assumptions. Volatile alkali is not a stimulant, in the ordinary sense of the term: it will produce a disagreeable impression upon the nose and upon the fauces; but, taken into the stomach in as large a quantity as can be swallowed, it has no effect upon the circulation.

In the first set of experiments, three dogs, about fifteen minutes after the bite, were severally treated, one with one drachm of spirit of turpentine; another with one drachm of spirit of ammonia, (what is this *spirit?*) a third had the same quantity of *spirit* of ammonia, with an ounce of alcohol poured down their throats, as soon as the symptoms of poison appeared, and died in less than an hour. A fourth case was bitten on the neck, like the two preceding, by the snake which had killed the first and third, besides a pigeon, and left to his fate. The object was to discover how long he would live without any remedy. Some slight symptoms came on, but went off shortly; and I have frequently heard since that he is still living." Other dogs were treated with arsenic, or spirit of ammonia. In one dog, excision of the part bitten was tried, with success.

Dr. STAUGHTON, of the United States, has communicated some observations on *Mania a Potu,* called also *Delirium Tremens,* from the tremors which either accompany or precede the mental derangement. This disease is little known in England, although not of unfrequent occurrence. In America, it appears that, in one alms-house, eighty-two cases occurred in two years. After hard drinking, the patient complains of nausea, head-ach, and chills; confusion of ideas, loss of appetite, restlessness, and want of sleep. About the same time, tremors come on, which are succeeded, sooner or later, by mania. The countenance exhibits strong marks of anxiety, though the eye is less wild and rolling than in a common phrensy. The behaviour of the patient is mild and docile. It is common, on entering his apartment, to find him propping up the wall, and importuning assistance to prevent its falling. He frequently changes the position of his bed, and goes about endeavouring to pick up money and other articles, which he supposes to lie scattered over the floor. He talks incessantly, seldom sleeps, and is in constant fear. Spontaneous cholera often relieves these symptoms. The operation of an emetic frequently brings away from the stomach a very peculiar fluid: it is thick, ropy, and viscid, like boiled tar. When a free discharge of this takes place, recovery generally follows. On dissection, the brain is found to be free from all appearances of inflammation. Slight effusion is seen in some few instances. The stomach, and sometimes the bowels and peritoneum, are inflamed to a very considerable degree. When the patient dies in convulsions, the cerebral vessels are gorged.

The treatment usually recommended is to exhibit opium and camphor; but emetics are preferred by this writer, who saw sixty-four patients cured by them out of eighty-two. The emetics sometimes require to be repeated. After taking them, the patient will be much comforted by some aromatic bitter,—such as a decoction of serpentaria, orange-peel, and quassia. Opiates are necessary, on account of the vigilance. The *tela araněi* is said to produce decided sedative effects; ten grains being equal to one grain of opium. The author considers that the situation from which the web is collected influences its quality. The tincture of hops is highly praised: in the dose of an ounce, "it soothes the watchful maniac into the sweetest slumbers."

Typhoid symptoms sometimes occur, wherein the emetic plan would be improper. Epispastics, rubefacients, brandy, opium, camphor, spirits of turpentine, and musk, are among the remedies which should be employed under these circumstances.

Our readers are already aware that the respectable physician, to whom we are indebted for the blessing of vaccination, has published some cases showing the efficacy of *emetic tartar ointment* in various disorders. Although the use of this remedy cannot be classed among the novelties of the day, yet the manner of its application,—not in the immediate vicinity of the *sedes morbi*, but to remote parts of the body,—is deserving of attention. As an example, we may here refer to the related cases of maniacal affections cured by rubbing the ointment into the inside of the arms; a particular account of which will be found in our review of the work.*

One of the causes of *sudden death*† has been elucidated by some dissections, which are scattered in contemporary Journals.

On the examination of a man who was supposed to have died suddenly of apoplexy, the *heart was found preternaturally softened*, and from whose surface a considerable quantity of blood had been effused. It was covered with a regular coating of coagulated blood, in some parts more than an inch in thickness. The pericardium contained a pint of serum, which corresponded in quantity with the weight of the coagulum. No rupture of the heart, or any of its vessels, could be discovered. The diseased had complained of slight difficulty of breathing.

Two other cases of sudden death are recorded. In one of these, the heart presented an appearance which, the writer says, he had not been accustomed to witness. The left side was in a loose and flattened state; whilst the superior part of the vena cava and right auricle appeared to be moderately distended with blood. The left auricle, and both ventricles, were in a complete state of collapse, and destitute of the smallest quantity of blood. The pericardium, which appeared much distended, had a bluish colour, and presented an evident degree of fluctuation, containing a quantity of serum and coagulated blood. The heart was ruptured at the anterior portion of the left ventricle.‡

In a third, no morbid appearances were found either in the abdomen or head, but the thorax was not examined.§

---

* *A Letter to Charles Henry Parry, M.D. &c. on the Influence of Artificial Eruptions.* By E. JENNER, M.D.—See *London Med. and Phys. Journal*, March 1822.

† *London Medical Repository*, April 1822.

‡ *London Medical and Physical Journal*, May 1822.

§ *Ib.* May 1822.

It may not be generally known that what is called sudden death, in cases of affection of the heart, is in fact *instantaneous;* whereas, in apoplexy, the patient dies stertorous or convulsed. Another example of this kind is, we think, afforded by the lamented death of the late Mr. Wilson, as appears from the following report.* This gentleman died while writing at his desk. On examination of the body, "the whole surface of the left lung adhered to the sides of the chest. Both lungs were loaded with blood and fluids, the left lung more especially; yet in neither was there any tubercular disease or induration. Both sides of the heart were empty, as also the thoracic aorta; and there was no disease in them except a slight thickening of the semilunar valves of that vessel. It therefore appears that, at the time of the death, the blood did not return to the heart, either from the lungs or general system. The vessels of the dura and pia mater were turgid with blood. Serous effusion to a considerable extent had taken place between the tunica arachnoidea and pia mater. The interior vessels of the brain did not appear distended, and there was not more fluid than usual in the ventricles. The coats of the right carotid artery were thickened where it merges from the cavernous sinuses, and this vessel was dilated in its course through that cavity."

Very discordant are the accounts respecting *iodine* as a remedy in bronchocele and scrofula. We have already noticed the testimony of Dr. del Carro and others in its favour. M. Gimele, of Paris, it appears, has successfully exhibited it, not only in these complaints, but in tumors of a doubtful description, in psoriasis, and other cutaneous diseases belonging to the order Squamæ. On the other hand, many practitioners of eminence have found it comparatively inert. Dr. Kennedy,† of Glasgow, administered it, in considerable quantity, to a female patient labouring under bronchocele, without benefit. Nine hundred and fifty-three grains of pure iodine were administered in the space of eighty-three days; but the case had also resisted a long employment of setons, and probably of other remedies previously employed.

In an account of some experiments made at Florence, to determine the action of the essential oil of the *lauro cerasus* on the animal economy, we find a suggestion, deserving of notice, respecting the advantage of administering it, instead of other preparations containing the hydrocyanic acid, in cases where the latter remedy is indicated. Unlike the distilled water of the plant and the pure acid, it always contains it in the same proportion. The great objection to the use of hydrocyanic acid, is the uncertainty of obtaining it of a uniform standard. Our correspondent, Mr. Bush, informs us, that he uses an emulsion of the bitter almonds, in which it is well known the acid is present. The essential oil of the bitter almond may be diluted by an expressed oil, and formed into an emulsion.

The communication of Mr. A. Todd Thomson,‡ on the benefit re-

---

* *Edinburgh Medical Journal,* April 1822.
† *London Medical Repository,* March 1822.
‡ *London Med. and Phys. Journal,* February 1822.

sulting from the application of *prussic acid* in cases of impetigo, is deserving of attention. The preceding remarks may suggest an economical mode of applying this remedy, with all the advantages resulting from uniformity of dose. It remains, however, to be ascertained, by experiment, whether, in this form, the same benefit would be derived.

We have often suspected that, in proportion to the advancement of medicine on the one hand, it made retrograde movements on the other. This fact is in no respect better evidenced than in the general neglect of medicines of unquestionable value, which, in the wisdom of chartered Colleges of Physicians, have unhappily been ejected from the Pharmacopœias. How frequently do we witness recoveries of patients in the hands of the herbalist, where the disease has resisted the skill of the most distinguished physicians. An instance of this is recorded in the person of an American consul, for whom the best medical skill was exerted in vain, for a troublesome tetterous affection of the eyes, which was ultimately cured by an Arabian doctor, who administered a plant called *le petit jubabe.* From an essay to determine the botanical name of the remedy thus designated, it appears to be the *sedum acre* of Linnæus; *la petite joubarbe,* or small house-leek.* The properties of this plant, as described by ALIBERT, in his Materia Medica, cannot be too generally known. It was for some time employed as an emetic.

The use of *phosphoric acid* in jaundice,† and of *tincture of opium* as an external application to polypus and tumors, merit notice.

From an essay on the *cornus circinata,* or mountain willow, by Dr. IVES, we learn that this remedy is in common use at Newhaven, as an astringent and an aromatic tonic, and is used as a substitute for Peruvian bark. It seems that it was first introduced by Professor Ives, in consequence of its having been successfully employed as a nostrum upon a patient labouring under a protracted disease of the bowels. From its superior efficacy to other means employed, he was induced to offer a reward for a specimen of the plant. From that time, which was about twelve years ago, he had been in the constant habit of prescribing it in cases indicating the use of vegetable tonics, and with such satisfactory results, that, for a number of years past, he had used more of it than he had of the Peruvian bark. The author has prescribed it in dysentery, after the bowels had been evacuated, and the febrile symptoms subsided; in protracted cases of diarrhœa, and in dyspepsia, accompanied with an enfeebled state of the stomach and bowels, with general debility. In diseases of this character, it is unquestionably a valuable remedy, and equal, if not superior, to any other tonic. Prof. Ives, of Newhaven, speaks highly of its efficacy in cholera infantum. In the preparation of ink or of black dye, it is superior to oak sumach or logwood, and but little inferior to that made with the Aleppo galls.

We have already referred to the efficacy of this remedy in the hands of Dr. HARRIS, under the article Intermittent Fever.

The *oil of croton,* as might be expected, continues to excite the attention of the medical public. Mr. ILIFF‡ gives a brief account of

* *American Medical Recorder,* January 1822.
† *London Med. and Phys. Journal,* May 1822.
‡ *London Medical Repository,* vol. xvi. p. 17.

fourteen experiments, in the greater number of which half a drop acted copiously on the bowels. Even in this small dose, it sometimes produces nausea and griping. In a case of apoplexy, three drops were given every two hours, until nine drops were administered, yet without effect, and the bowels were ultimately opened by ten grains of calomel. A hearsay correspondent, in the same Journal, *heard* of some gentleman who had given, without the smallest success, ten minims of the oil obtained from the Apothecaries' Company. If this be really the fact, we suspect the oil must have been bad; the possibility of which should be kept in the view of every experimenter. We shall not, however, gain much by the introduction of a new purgative, unless it can be shown to possess more efficacy in the cure of disease than others of the same class. We shall then hail its introduction with a degree of satisfaction that we have not yet experienced at the intelligence respecting it.

Dr. NIMMO, in an essay communicated to the Journal of Science,* entertains the most sanguine hopes of its virtues. We have seen so much of the beatific visions of medical men, on the introduction of new remedies, that we do not easily become the converts of speculations of this kind. The Doctor assures us, that he has administered it in an hundred doses; "that in not more than three or four cases was vomiting produced;" purging speedily took place, and was rarely accompanied with griping. Among the cases in which it was administered with success, was a case of ascites. In delirium tremens, it was found to be a powerful auxiliary to opium. It has proved beneficial in jaundice from obstructions or spasms of the bowels, and in tympanitis intestinalis. Lastly, we are told that, in a case of excessive corpulence, with the most alarming symptoms of determination to the head, amounting almost to apoplexy, it produced the most signal benefit; and, without having occasion to let blood, all was effected that could have been expected from the latter operation.

We are indebted to this author for a useful analysis of the oil, from which it appears that it is a binary compound, and that the medicinal quality resides in an extractive matter, separable by alcohol. This principle exists in the proportion of 45 to 55 parts in 100, constituting nearly one half. The remainder is like olive-oil, insipid, and wholly inert. Both these principles may be obtained in combination, either by expression or from the seeds, by immersion in ether or in purified oil of turpentine; but, as the medicinal quality resides in the extractive matter, the best method of obtaining it seems to be to make a tincture of the seeds in alcohol, which, mixed with mucilaginous substances and water, in the form of a draught, affords a more convenient vehicle for its administration; and is supposed to be more speedy in its operation, in consequence of its immediate diffusion over the stomach and intestines.

The subject is also taken up by some others; and particularly by Dr. CARTER,† who tried it in seventeen cases, with satisfactory results as to its purgative operation. One case of dropsy was considerably

* See also *London Med. and Phys. Journal*, vol. xlvii. p. 378.
† *London Med. Repository*, February 1822.

relieved by it; but the patient remained under cure when the paper was written.

Among the prophylactic remedies, *belladonna* is recommended, and apparently on good authority, to secure the constitution against scarlatina. Assumed facts of this description have, however, been so often thrust down the throats of the credulous, that we look upon every thing of the kind with distrust. The *scarabæus melalontha* and *cantharides* have been severally recommended as preservatives against hydrophobia.

One of the most extensive ravages of the latter disorder which, of late years, it has been our painful duty to record, occurred in the practice of Mr. BRERA, a well-known Italian physician. Although this event took place some years since, the account has but recently come to hand, through the channel of the *Annali di Medicina*;* we shall, therefore, notice it both here and in our Critical Analysis, conceiving it to be fraught with matter for useful reflection. Of thirteen persons bitten by a rabid wolf, nine died. As soon as the rabid nature of the animal was clearly ascertained, by the death of the first of these unhappy victims, the remainder of those bitten were subjected to prophylactic treatment of various kinds. Four escaped the disease; and, as these four *only* were subjected to the *same* vigorous treatment, a strong presumption may be derived in favour of the efficacy of the remedies employed; for the particulars of which we refer to our analytical department.

Mr. BATTLEY, whose attention has been for many years directed to preparations of *opium*, considers the resinous part alone to be that which produces constipation; having witnessed this effect in more than twenty cases. This principle should therefore be excluded when the sedative property only is required. The French chemists, observing that one of the principles of opium, known by the name of *salt of Derosne*, which is improperly called also narcotine, produces a stupor differing from real sleep, deprive it of this irritating and pernicious material, by treating a thin watery extract with repeated agitations in ether, until the whole of the saline matter is dissolved in the latter.

Thus far we have endeavoured to concentrate into a narrow focus the principal facts which have been published on Medicine and the Materia Medica, within the period contemplated in this Essay. Some others might have been adverted to with propriety; but we are reminded, at every step, that we are trespassing upon the boundaries of other departments of our Journal: we shall therefore confine our remaining observations to a cursory notice of a few facts which lie before us on Surgery and Midwifery.

### II. CHIRURGERY.

Unless we are to estimate the merit of an operation by the mere difficulty which attends its performance, we should place in the foremost rank of improvement the cure of an ovarian dropsy by extirpation of the sac, after the evacuation of its contents. This case, related by Dr.

* August 1821.

NATHAN SMITH, we have already recorded,* so as to render any further particulars unnecessary in this place.

The operation of tying the superior thyroid artery, as recommended by Sir W. BLIZARD, in bronchocele, has been performed by Dr. JAMESON, of Baltimore. The patient had been affected, about twenty years, with a very considerable enlargement of the thyroid gland, and a tumor on the left side of the neck, of considerable size, and in some degree distinct from the original tumor. The tumor, a few weeks after the operation, which had previously been very painful, could now be handled without uneasiness; and, at the time of writing the account, was considerably less in dimension. The author thinks that, if the vessel on the opposite side were removed, the tumor would disappear.

Dr. KENNEDY has supplied some observations on the several modes of treating *Bronchocele.*† That by seton, as recommended by QUADRI and other respectable surgeons, he has put to the test of experiment in two patients, without effecting relief. We advert to this circumstance, not as affording conclusive evidence against this treatment, but conceiving that the practitioner should be in possession of the facts on each side of the question. Nothing is so dangerous as extravagant notions of the efficacy of therapeutic measures. The least failure produces a disappointment in proportion to the previous expectation, and often leads to the total abandonment of, perhaps, a valuable remedy. The ligature of the thyroid artery passes also under review. The writer, in the teeth of an instructive case from the Medico-Chirurgical Transactions, which he quotes, considers it to be of doubtful efficacy; but thinks it may be tried, especially if the tumor be firm, and seems to have originated from excessive arterial action. The most interesting part of this paper relates to the excision of the tumor, which, although rarely performed, has been considered to be practicable by many distinguished surgeons; and some instances of successful result are recorded, as those of the Savoyard barber, Giraudy, Theden, Vogel, Treytag, and Dessault. The author has compressed into a narrow focus the names and sentiments of many advocates on each side of the question. Foremost amongst those who favour the operation stands Celsus, (who says "Potest adurentibus medicamentis curari sed scalpelli curatio brevior est." Dionis, Brouzet, La Faye, Dessault, Foderé, Leveillé, Hevin, Dupuytren, Mr. Allan Burns, and Mr. Samuel Cooper, advise it under modified circumstances. On the other hand, many, deterred by the dread of hemorrhage, proscribe the operation as being rash and pernicious: these are, Paul of Ægina, Albucasis, Severinus, Palfin, Petit, Radel, Pelletan, Haller, Lassus, and the majority of English surgeons. The author has, however, supplied us with two cases, which sufficiently demonstrate the practicability of the operation, although they both terminated fatally. One was performed by DUPUYTREN, which our limits will not permit us to record. In a boy, operated upon by Dr.

* *London Medical and Physical Journal,* April 1822.
† *London Medical Repository,* March 1822.

KLEIN, of Stuttgardt, the tumor occupied all the left side of the neck from the ear to the larynx, and stretched downwards to the third rib. "Blood-vessels, thick as one's finger, ramified over its surface. From its weight, more than its adhesions, it was almost immoveable. At the base, it was six inches in breadth, and five in depth. Its transverse diameter measured sixteen; its perpendicular, eleven inches and a half. In some parts it was knobbed; in others, the pulsations of its deep-seated arteries were strong and visible.

"*Operation.*—Two oval incisions, three fingers' breadth distant from each other in the centre, were rapidly made in the tumor. The cutaneous veins, which were greatly dilated, threw out half a pound of blood before they could be compressed. The tumor was now denuded by dissecting laterally from the first incision; when the operator, drawing it forwards, and using his finger on the handle of a bistoury, separated it from the left carotid artery, the larynx, and trachea. The excision was completed in a minute and a half; but, to the surprise of the surgeons, not a drop of blood issued from any vessel, except what was poured out at first by the subcutaneous veins. The patient was now discovered to be insensible: he was dead.

"*Dissection.*—The left carotid artery, and corresponding jugular vein, the par vagum, and trachea, had escaped being injured; but all the vessels of the encephalon and dura mater were distended with blood. There was much serum in both ventricles, and under the arachnoid of the right ventricle. From these appearances, Dr. Klein concludes that the boy died of apoplexy, induced 'par la révolution que l'opération lui avait causée.'"

In neither of the cases related, the patients died from loss of blood. The author suggests that, in the case of Dupuytren, it was owing to injury inflicted upon the great sympathetic nerve; an opinion we could abundantly support by the histories of operations in these parts, but our space reminds us not to enter so fully upon the subject. Division of the respiratory and cardiac nerves in the neck would be followed by immediate cessation of the functions of the heart and lungs. A supposition of this kind offers a more rational mode of accounting for the sudden death of the child, than that he died of apoplexy. Upon this view of the subject, it will be evident how important it is to make accurate observations of the parts after an unsuccessful operation. From Dupuytren's case we learn, that the right recurrent laryngeal nerve was cut, and included in the ligature. The great branch of the hypoglossal, which anastomoses with the cervical pair, was in the same state. In the left side, the first of these nerves was not divided, but was putrid and livid in its superior part. The pneumo-gastric nerves were neither injured nor displaced. Klein simply tells us, on this subject, that the par vagum had escaped being injured. It may be useful to add, that, in a case of dissection, accurately related by the late Mr. ALLAN BURNS, the par vagum and sympathetic nerves were, on both sides, pressed on the tumor. In other instances, however, hemorrhage has been the cause of failure. In a fatal case, under the hands of DESSAULT, a terrible hemorrhage obliged him to desist; he then passed several ligatures round the base of the tumor; but violent spasms were excited, and the woman died. In two other cases referred to, the patients died from uncontrollable hemorrhagy.

Mr. Hayden, of Baltimore, has furnished a paper on the devastation of the teeth and gums, under the title given to it by the older French dentists, *conjoined Suppuration of the Gums and Alveoli.** The main object of this communication is to show, in answer to Mr. Koecker, who had previously written on the same subject, that the disease was not only known, but, after having been previously noticed as a scorbutic affection, as Stomacace, had been accurately described, as far back as 1728, by Fauchard; and, again, by Lecluse, Courtois, Ricci, Jourdain, Bounon, Bourdet, and Fox. It has also been mentioned by Hunter.

Mr. Maunoir, of Geneva, has communicated a case in which the temporary ligature of the carotid was employed for the cure of a tumor, supposed to be aneurism. The artery was tied near its bifurcation, with the interposition of a pledget of lint. On the third day, the operator relieved the artery from its pressure, and withdrew the pledget. The vessel was found perfectly closed, no pulsation being felt in it above the part tied. The tumor was about the size of a hen's egg, and fluctuating. It had existed six months, and almost disappeared upon being pressed with the finger; but immediately returned on the removal of the pressure. The absence of pulsation rendered the nature of the swelling doubtful, but it was considered to be an aneurism of a vessel embedded in the parotid. The patient suffered from continual and acute pain in the globe of the left eye, and behind the ear of the same side. The jaws were fixed, as in trismus. Pressure of the tumor, by means of a permanent apparatus, was first tried, and borne with great constancy by the patient, notwithstanding the pain it occasioned. Artificial cold was applied, by which the symptoms were calmed, but the tumor neither lessened nor varied in consistence. For some weeks after the application of the ligature, the tumor seemed to diminish in bulk; but at the time of writing had regained its original size: the patient was, moreover, weak and emaciated. The only permanent advantage gained was sleep and freedom from pain; of the former of which he was deprived before the operation. The editor of the *Annali di Medicina*,† from whom we derive this account, suggests that the case was anastomosing aneurism, and that, although the trunk of the artery was closed, yet the tumor was afterwards supplied by the anastomosing vessels.

The opinions of Mr. J. Bell as to the necessity of extirpating anastomosing aneurisms, has been already refuted, by the cases of this disease in the branches of the carotid artery, already before the public. Another instructive example of the same kind has been communicated by Mr. Pattison, the professor of anatomy in the university of Maryland; where the ligature of the carotid terminated favourably, although, from the extent and situation of the tumor, a successful result seemed very

* *American Medical Recorder*, January 1822. † 1821.

problematical. The tumor was very large; its central point occupied the situation of the antrum highmorianum; but, the walls of this cavity having been destroyed, it passed from thence in every direction upwards, into the orbit, protruding the eye nasally, it passed into the nostril of the left side, which it completely filled, and, pressing on the septum-narium, it gave a general character of distortion to the nose. This tumor was, however, most prominent in a direction outwards. The anterior wall of the antrum having been previously removed by an operation, there was nothing opposed to its passage, except the small facial muscles; and their forces, although they might have a tendency to prevent its direct growth outwards, could have little effect in restraining it from growing in a direction outwards and backwards. The symmetry of the left side of the countenance was completely destroyed. The tumor, which in size was nearly equal to the head of a new-born child, extended from the left margin of the nose to the line which marked on the neck the tracheal margin of the sterno cleido mastoideus muscle. When the disease was examined as it appeared in the nostril, the first impression produced was that of a polypus in the antrum.

Two operations had been previously performed under this notion. The first was by ligature and forceps. At length, however, the cheek began to swell; it was supposed that the polypus had originated from the antrum, and an operation was performed with the view of extirpating it, which consisted in removing, with the trephine, the anterior wall of the antrum; and, thus having exposed the tumor, an attempt was made to cut it out. The bleeding was, however, such as soon to put a stop to the operation. The disease had existed nearly eight years, in the course of which time hemorrhage frequently took place to a tremendous degree.

The cure of a variety of this disease, the *Nævus Maternus*, by excision, is now very common. Mr. LANGSTAFF has frequently removed it in this manner. The hemorrhage has always been considerable, although the healthy skin was removed as far from the disease as prudence dictated. The principal part of a lip was removed in a child, three months old, affected with nævus, which, from its magnitude and discoloration, gave the child an hideous appearance. The operation was performed as in hare-lip; the labial arteries, as may be supposed, bled very freely. The author has succeeded in destroying the smaller nævi by potassa fusa. The sloughs were thrown off without hemorrhage. In removing nævi from the tip of the nose, the bleeding is described to be very perplexing, from the great vascularity of the part, and the impossibility of removing a sufficient quantity of the sound integuments.

The Quarterly Journal of Foreign Science* records the following case of *Spina Bifida*, treated according to the method of Sir ASTLEY COOPER; communicated by M. RICHT, of Berlin.

"The wife of a labourer was delivered, at the full period of gesta-

* April 1822.

tion, of a healthy-looking child, on the lower part of whose spine was a transparent swelling, of the size of a walnut. The child seemed paralysed in the lower extremities, and was subject to involuntary discharge of the urine and feces. On examination, the parts were found to have all the characters of spina bifida; the skin forming a bladder, in which fluctuation was distinctly felt. The lower extremities seemed to be well nourished, notwithstanding the paralysis. The little patient had great difficulty in sucking. After evacuating by the needle a small quantity of lymph, moderate pressure was applied, and *the midwife who attended was ordered to repeat the puncture and the pressure!* In four days, the physician found the tumor considerably diminished, the skin very hard, and a great number of minute blood-vessels ramifying through it. The paralysis of the lower extremities seemed to give way, the involuntary evacuations ceased, and sucking was more easily performed. The treatment *was ordered* to be repeated. The midwife having been called to another patient, the mother was instructed to perform the puncture and the pressure! But the good woman, in haste to evacuate at once the contents of the tumor, made a considerable opening. Spasmodic fits soon supervened, and the infant expired about twenty-four hours after the puncture had been made."

Mr. CAMOIN,* of Odessa, describes an operation for the *Extraction of Stone by the Rectum*, performed by him in the hospital of that city. After having introduced a sound, as in the ordinary lithotomy, the fore-finger of the left hand, in a state of supination, was introduced into the rectum, on which the flat side of a bistoury was passed, as recommended by Mr. SANSON. An incision was then made from within outward, in the direction of the raphé, through the lower part of the rectum and sphincter ani; by means of which the prostate gland could be easily explored. The finger then being carried beyond the gland, the groove of the sound was felt through the rectum and lower part of the bladder, the point of the bistoury was passed into it, and an incision was made about an inch and a half in length. The liquid contents of the bladder having escaped, satisfied the operator that he had penetrated its cavity; upon which he withdrew the sound, and introduced the forceps. Some little difficulty was experienced in finding the stone, in consequence of its adherence to the neck of the bladder. It was, however, detached by the fore-finger, seized with the forceps, and easily extracted. Not more than a tea-spoonful of blood was lost. The operator has such confidence in this method, that he professes his determination, in future, always to adopt it; but, in consequence of some impediment offered to his incisions by the constriction of the sphincter, suggests the expediency of previously introducing a dilating gorget into the rectum, which, by keeping back its posterior surface, and by enlarging the anus, allows greater space for the proceedings of the operation.

Mr. WALLACE,† of Dublin, records an example of tracheotomy,

* *Journal Complementaire*, March 1822.
† *Quarterly Journal of Foreign Science*, April 1822.

successfully performed in a child, whose respiration was almost suspended in consequence of inflammation at the upper part of the larynx, by swallowing boiling water. The trachea, after the operation, would not admit a canula, which caused pain and spasm. The narrator, therefore, removed a portion of the wings of the trachea, which answered extremely well. It became necessary to keep asunder the lips of the external wound, which was done by a small canula, and every prospect existed of the child's recovery at the time of writing.

In a case of *Croup*, tracheotomy proved unsuccessful in the hands of Mr. BRICHETEAU, although the pseudo membrane had been removed from the trachea.* As this membrane frequently lines the bronchia as well as the larynx, it must not be expected that we can always command success, were no other cause of death present to defeat our efforts.

Many of our readers will recollect to have seen, in our Number for January 1821, the important narratives of the cases of *Artificial Anus*, formed by an incision into the sigmoid flexure of the colon. Mr. PRING, one of the operators on this occasion, has communicated the sequel of his case.† It will be obvious that, when this method is resorted to under circumstances of obstruction and fatal disease, all that can be expected from it is a prolongation of life. Such was the effect with the patient: she survived the operation a year and four months, but at length fell a victim to the disease in the rectum, which had rendered it necessary. On examination after death, "the principal disease commenced at the top of the rectum: this gut was, from its connexion with the colon, almost of a cartilaginous firmness; it was very much thickened, and its calibre, of course, greatly diminished. Posteriorly, the rectum was firmly adherent to the sacrum: at about its middle portion, in front, it was destroyed, for the space of perhaps two inches in length, by ulceration. Below this part it preserved the cartilaginous structure, and was so much thickened, that its canal barely admitted the introduction of an urethra bougie. The rectum was not continued to the natural anus, but appeared totally destroyed by sloughing and ulceration to the extent of two inches before it reached this part.

A memoir has been published by an Italian surgeon,‡ to show that, in the cure of *Varicose Veins*, excision of a portion of the vessel is preferable to the ligature. This, however, was known to CELSUS, and has been long since tried by some of our London surgeons; but abandoned for the safer operation of dividing the varix under the skin. By leaving entire the superincumbent skin, less danger exists of that inflammation which has so frequently proved fatal. The mode usually employed is to introduce a knife, with its flat surface parallel to the skin, between the skin and the varix, till it is carried across the latter,

* *London Med. and Phys. Journal.*

† *London Medical and Physical Journal,* February 1822.

‡ *Della Maniera piu atta a Curare radicalmente le Varici ed Impiagamenti Varicosi dell' Estremità inferiori.* Memoria di RANIERI CARTONI. Pisa, 1821.

when the cutting edge is to be turned downwards, and the vein divided. The knife should be curved, with its cutting part on its convex edge.

A paper on this disease has been supplied by Mr. LLOYD,* of Falcon-square. This gentleman is of opinion, that the division of the vein under the skin is the most effectual operation, and attended with less danger. He very properly remarks, that the cure of the disease does not depend on the obliteration of the trunk of the vessel, since the varicose state of the limb is not thereby removed; nor is the obliteration itself essential to its cure. It is to the inflamed and morbid state of the varix itself we are to look for the cause of the disease, attendant on the ulceration of the integuments. Hence the section of the diseased vein or veins should be effected in the immediate vicinity of the ulceration. The surgeon should, previously to operating, ascertain that the vein is not only varicous, but painful and tender to the touch. Sometimes one or two only are discovered to be in this state; in which case, it is sufficient to divide them. "When the disease is of long standing, it often requires a very attentive examination to discover the diseased varices, unless they are in a very tender condition, as the cellular substance surrounding the varix becomes so much thickened, and the skin so much condensed and deprived of its elasticity by repeated attacks of inflammation, that they no longer present outwardly, as when simply varicose; but the leg at the diseased part has a smooth surface, and appears as if the skin was tightly stretched over it. A careful examination will, however, generally enable us to discover the varix, by a finger placed above it, while, with the other hand, the blood is impelled towards it from the vein above and below. This, together with the pain and tenderness, will sufficiently indicate the situation on which it will be most advisable to perform the operation. In other cases in which the veins have lost their prominent appearance, their situation is easily ascertained by the thickening of the cellular substance on each side of them, which creates a feeling as if they were lying in a cavity with hardened edges. The one to be divided is determined, as in other cases, by the comparative tenderness on pressure, and without any reference to its being above or below the ulcer."

These views respecting the morbid state of the veins are not new, but are not generally taken by practitioners. We have frequently seen varices cured by leeches applied to the affected portions of the vessel.

An accession has been made to operative surgery, by new instruments for *Nasal Polypus*, and for the *Extirpation of the Mouth and Neck of the Uterus*, when attacked with schirrus or excrescence. The latter is the invention of Dr. CANELLA. That these parts might be amputated with safety to the patient, has never, we think, been denied; but the practicability of the operation, in the natural position of the uterus, has been hitherto considered problematical. DUPUYTREN, we remember, in one case, grasped the mouth of the uterus with forceps, and, having succeeded in bringing it to the orifice of the vagina, removed it with his scissars. The disease, however, returned, and another portion was

* *Quarterly Journal of Foreign Science.*

removed; yet not of sufficient depth for the cure of the patient, who was ultimately obliged to undergo a long and painful application of the caustic, conveyed to the part. An advantage resulting from the contrivance under consideration, is that, whatever be the depth at which the uterus may be situated in the vagina, the excision may be performed with equal ease and safety.

A case of *fatal Mortification of the Cheek*, in which pyroligneous acid was employed, by Dr. MOORE,* with complete success, to correct the discharge, is deserving of attention. Under a course of mercury for hydrocephalus, the left cheek had become much swollen, inflamed, and painful; the ptyalism was moderate, and the mouth generally was not very sore, but the pain and swelling of the cheek continued for some days to increase, notwithstanding all means made use of for its relief. A black spot, as large as a sixpenny piece, soon appeared, about an inch from the angle of the mouth, which increased to the size of a dollar, destroying all the parts between it and the mouth. The dead matter shortly melted down into a mass of putrefaction, which rendered the atmosphere of the apartment so excessively offensive, that the nurse found it impossible, without serious inconvenience, to pay the child those attentions her situation demanded. A fermenting poultice had been directed in the first instance, but they found difficulty in keeping it applied. Having before experienced the powerfully antiseptic properties of the pyroligneous acid, lint was directed to be wetted with it, and applied to the mortified part as often as might be found necessary to correct the fetid odour. After its application, there was not the least unpleasant odour to be perceived on the closest examination.

We have transferred to our Medical Intelligence an account of some highly interesting experiments, made by Dr. WALTHER, to determine the possibility of re-uniting the osseous disc removed by the operation of the trephine. The affirmative is indisputably proved by the facts stated: indeed, it was demonstrated several years since by Dr. MERREM, an account of which was published in his Surgical Observations.†

Mr. RENAUD's extraction of a fork from the stomach by gastrotomy, and the successful removal of a portion of the lower jaw from its articulation, by Professor GRAFE, of Berlin, evince a dexterity on the part of the operators which is highly creditable to them.‡

### III. MIDWIFERY.

The communications on this subject are very few. Dr. POWER§ relates a case of protracted labour, with convulsions and mania. The convulsions were abated by bleeding, but were succeeded by violent maniacal excitement. The os uteri was fully dilated, but the womb had ceased to contract. The writer commenced a vigorous friction on the

---

* *New York Repository*, January 1822.

† *Animadversiones quædam Chirurgicæ Experimentis in Animalibus Factis illustratæ.* Gressæ, 1810.

‡ *London Medical and Physical Journal.*

§ *London Medical Repository*, January 1822.

belly, and in a few minutes a decided contraction of the uterus took place, as was proved by pain and a tense, hard state of the abdominal uterine tumor; the head of the child being propelled, with much force, into the os externum. It was, however, deemed expedient to deliver the patient by the forceps; after which she sunk into a deep sleep, which lasted several hours. The efficacy of abdominal frictions, in cases of protracted labour, is highly lauded.

A fatal case of *Laceration of the Cervix Uteri during Labour*, is recorded by Mr. ASPRAY. The head and arm had descended through a narrowed pelvis, and was within reach of the forceps, which the patient would not allow to be employed, until it was found that the head had receded so far that the instrument could not be used. The child, which was dead, was now turned and delivered; but the mother shortly expired.

Considerable experience in the treatment of labour induces us to believe that the use of the forceps is often most improperly delayed; and to this alone are we to ascribe the death both of the mother and child in many cases, who might have been saved by more decision on the part of the practitioner.

The Number of our Journal for April last supplies us with another fatal case of labour, showing the danger of delay in the use of instruments. The highly respectable practitioner to whom we are indebted for it found his patient, after having been two days in labour, with the vertex of the child presenting at the upper orifice of a pelvis, *whose diameter from before backwards was unusually short*, "and there appeared but little hope that the obstacle could be overcome by any efforts which the remaining strength of the mother might be equal to: *she was not, however, so completely exhausted as to justify an immediate resort to the operation of cephalotomy.*" At what hour of the day this examination was made, does not appear; but, at three in the afternoon, on a second and more minute examination, the parietal bones were found wedged between the pubis and base of the cranium. The operation was now performed, the child delivered; but, in about ten days afterwards, the mother died.

In illustration of the same opinion, we may adduce another fatal case, related by Mr. ALEXANDER, of Dumferline. The impediment in this patient arose from the locking of the heads of twins. This patient had been in labour three days, under the care of a midwife, before the author was called. The body, feet, and arms of one child were delivered, and the head of another was in the pelvis. The labour was too far advanced to turn, and the only chance which could be afforded to the patient was the destruction of both children. This was effected, and she was delivered, but subsequently died. It is not unreasonable to conclude, that, had this gentleman been called in at an earlier stage of the labour, the unfortunate presentation would have been soon discovered and remedied. Even an hour gained might have proved the salvation of this woman's life.

